# Supplementary material for: Nucleosome plasticity is a critical element of chromatin liquid–liquid phase separation and multivalent nucleosome interactions
Source: Nat Commun. 2021 May 17;12:2883. doi: 10.1038/s41467-021-23090-3 (PMC8129070; doi:10.1038/s41467-021-23090-3)
Supplement: Supplementary file 1 — Supplementary Information [file 41467_2021_23090_MOESM1_ESM.pdf]

Supplementary Information for: Nucleosome plasticity is a critical element of chromatin liquid–liquid phase separation and multivalent nucleosome interactions

Stephen E. Farr[1], Esmae J. Woods[1], Jerelle A. Joseph[1,2,3], Adiran Garaizar[1], and Rosana Collepardo-Guevara[1,2,3,*]

[1]Maxwell Centre, Cavendish Laboratory, Department of Physics, University of Cambridge, J J Thomson Avenue, Cambridge CB3 0HE
[2]Yusuf Hamied Department of Chemistry, University of Cambridge, Lensfield Road, Cambridge, CB2 1EW
[3]Department of Genetics, University of Cambridge, Cambridge, CB2 3EH
[*]rc597@cam.ac.uk

Contents

Supplementary Methods . . . **2**
Chromatin Multiscale Methodology . . . 2
Computational implementation . . . 2
Chemically specific model . . . 2
Chemically-specific model potential energy function . . . 2
Rigid base-pair potential forces and torques . . . 3
Model building & implementation of the chemically-specific model . . . 4
Chemically-specific model parameters: Hydrophobic interactions . . . 5
Chemically-specific model parameters: Electrostatic interactions . . . 5
Minimal Model . . . 5
Minimal model potential energy function . . . 5
Anisotropic potential forces and torques . . . 6
Model building & implementation of the minimal model . . . 7
Fitting of the minimal model parameters from chemically-specific model simulations . . . 7
Parameters of the minimal model potentials . . . 8
Simulation protocols . . . 9
Debye-length replica exchange simulation method . . . 9
Details of chemically-specific model 12-nucleosome chromatin simulations . . . 9
Details of the chemically-specific model nucleosome unwrapping potential of mean force simulations . . . 9
Details of the chemically-specific model nucleosome-nucleosome potential of mean force simulations . . . 10
Estimation of radius of gyration from simulations with our minimal model . . . 10
Details of the minimal model nucleosome unwrapping potential of mean force simulations . . . 10
Direct coexistence simulations . . . 10
Additional Algorithms . . . 10
Quaternion to rotation matrix . . . 10
Computation of helical parameters . . . 11
Analysis of the chemically-specific model simulations . . . 12
Orientation dependent nucleosome-nucleosome interactions . . . 12
Sedimentation coefficient . . . 12
Amount of unwrapped DNA . . . 13
Nucleosome valency . . . 13

Nucleosome–nucleosome contacts . . . 13
Analysis of minimal simulations . . . 14
Radius of gyration . . . 14
Estimation of liquid-network connectivity . . . 14
Supplementary Notes **14**
Timescale difference between chemically-specific and minimal model . . . 14
Nucleosome formation . . . 14
Persistence length of DNA . . . 15
Comparison with force-spectroscopy experiments of mononucleosomes . . . 15
Force-extension simulations of oligonucleosomes . . . 15
Results for a longer NRL . . . 16
Supplementary Tables **17**
Supplementary Figures **20**
Supplementary References **29**

Supplementary Methods

Chromatin Multiscale Methodology

We develop a multiscale method to investigate self-organization and liquid–liquid phase separation (LLPS) of chromatin at a range of resolutions, and to link atomistic properties with the emergence of collective behavior (Figure 1 and Supplementary Figure 1). In previous work, we performed simulations at level 1—all-atom simulations of a 211-bp nucleosome in explicit solvent with ions—that enabled the differentiation between histone globular domains and the histone tails [1]. Information was distilled from level 1 to develop level 2—our 'chemically-specific' coarse-grained model, which considers amino-acid and DNA base-pair level resolution. To reach the system sizes needed to investigate LLPS of chromatin arrays, further coarse-graining was developed to produce level 3—our 'minimal' coarse-grained model at nucleosome level resolution.

Computational implementation

We implement both the chemically-specific and minimal model in the molecular dynamics (MD) package 'Large-scale Atomic/Molecular Massively Parallel Simulator' (LAMMPS; https://lammps.sandia.gov/) [2]. Finite size ellipsoidal particles—essential for the implementation of orientation dependent potentials—from the `ASPHERE` package are used to represent coarse-grained segments of chromatin. Additionally, the `RIGID` package is used to enable the building of composite rigid bodies.

We use LAMMPS stable version 3rd March 2020 compiled with our custom code (see Code Availability section of the main text). All simulations were performed on the Cambridge Service for Data Driven Discovery (CSD3).

Chemically specific model

Chemically-specific model potential energy function

The total potential energy function for the chemically-specific coarse-grained model is,

$$E = E_{\text{Protein bonds}} + E_{\text{DNA bonds}} + E_{\text{Electrostatic}} + E_{\text{Hydrophobic}}. \tag{1}$$

Both the electrostatic and hydrophobic terms have a cutoff distance—beyond which the energy goes to zero—which is necessary for efficient computational implementation. The protein bonds are a standard harmonic interaction,

$$E_{\text{Protein bonds}} = \sum_{\text{all bonds}} \frac{1}{2} k (r - r_0)^2, \tag{2}$$

where $k = 20\,\text{kcal/mol/Å}^2$ is the spring constant and r_0 is the (bond dependent) equilibrium bond length.

The DNA bonds are modeled by the Rigid Base Pair (RBP) potential [3–7], which represents DNA base-pairs (bp) as rigid planes and the interactions between two adjacent base-pairs in terms of harmonic deformations of six helical parameters (three displacements: shift, slide, rise, and three angles: tilt, roll, twist).

$$E_{\text{DNA bonds}} = \sum_{\text{all bonds}} E_{\text{RBP}} = \sum_{\text{all bonds}} \frac{1}{2} \boldsymbol{\Delta\phi}^{\intercal} \mathbf{K} \boldsymbol{\Delta\phi}, \tag{3}$$

$$\boldsymbol{\Delta\phi} = (\boldsymbol{\phi} - \boldsymbol{\phi}_0);$$

$$\boldsymbol{\phi} = (\text{shift}, \text{slide}, \text{rise}, \text{tilt}, \text{roll}, \text{twist}),$$

where $\boldsymbol{\phi}$ is a 6-dimensional vector of the 6 DNA helical parameters, $\boldsymbol{\phi}_0$ are the equilibrium values and $\mathbf{K}$ is the 6×6 stiffness matrix. This model is DNA sequence dependent so $\mathbf{K}$ and $\boldsymbol{\phi}_0$ are dependent on the base pair step. The helical parameters are determined using the SCHNAaP method [8]. The DNA–DNA bonds use the RBP potential parameters from the Orozco group [6, 7].

Electrostatics are modeled by the Debye–Hückel potential, which accounts for screening due to an implicit monovalent salt solvent,

$$E_{\text{Electrostatic}} = \sum_i \sum_{j<i} \begin{cases} \frac{1}{4\pi\epsilon_0\epsilon_r}\frac{q_i q_j}{r} e^{-\kappa r}, & r < r_c, \\ 0, & r_c \leq r, \end{cases} \tag{4}$$

where ϵ_0 and ϵ_r are the permittivity of free space and the relative permittivity respectively, q_i and q_j are the charges of particles i and j, r is the distance between the interacting pair of particles, κ is the inverse Debye length and r_c is the cut-off distance (necessary for efficient computational implementation). Electrostatic interactions are considered between all charged particles within the cutoff distance.

To describe short-range non-bonded interactions, we follow the work of Dignon et al. [9] and use their version of the Miyazawa-Jerningan potential [10] with the functional form and parameters of Kim and Hummer [11]. This consists of a sequence-dependent hydrophobic attraction between specific amino acid pairs (Kim-Hummer model [11]), and a standard Lennard-Jones interaction [9].

$$E_{\text{Hydrophobic}} = \sum_i \sum_{j<i} \begin{cases} 4\epsilon_{ij}\left[\left(\frac{\sigma_{ij}}{r}\right)^{12} - \left(\frac{\sigma_{ij}}{r}\right)^{6}\right] + (1-\lambda_{ij})\epsilon_{ij}, & r < 2^{1/6}\sigma_{ij} \\ \lambda_{ij} 4\epsilon_{ij}\left[\left(\frac{\sigma_{ij}}{r}\right)^{12} - \left(\frac{\sigma_{ij}}{r}\right)^{6}\right], & 2^{1/6}\sigma_{ij} \leq r < r_c, \\ 0, & r_c \leq r; \end{cases} \tag{5}$$

where ϵ_{ij} is the dispersion energy, σ_{ij} is the distance at which a standard Lennard-Jones interaction is zero, λ_{ij} is a hydrophobic parameter, r_c is the cutoff distance (numerical value different to that for the electrostatic potential) and the sum is over all pairs of particles. Note that when $\lambda = 1$, we recover a standard Lennard-Jones potential, and when $\lambda = 0$, we obtain a purely repulsive Weeks-Chandler-Anderson (WCA) potential. Each amino-acid pair has unique values for ϵ_{ij} and λ_{ij}. σ_{ij} is defined using the combination rule $\sigma_{ij} = (\sigma_i + \sigma_j)/2$, where σ_i, σ_j are the van der Waals radii of particles i,j respectively. For interactions involving DNA, $\lambda_{ij} = 1$.

The two non-bonded interactions $E_{\text{Electrostatic}}$ and $E_{\text{Hydrophobic}}$ are turned off between directly bonded beads.

A summary of all the chemically-specific model parameters is given in Supplementary Table 9.

Rigid base-pair potential forces and torques

Typically the RBP model is used in Monte Carlo simulations, which only require the potential energy and not its derivatives. To implement the RBP potential in MD simulations, forces and torques must be defined. Following the method of Fathizadeh et al [12], the force in the kth direction on a base-pair, due to the RBP potential E_{RBP} is,

$$F_k = -\frac{\partial E_{\text{RBP}}}{\partial r_k} = -\sum_{i=1}^{3} T_{ik} \sum_{j=1}^{6} K_{ij}\Delta\phi_j, \tag{6}$$

where $\mathbf{T}$ (see Eq. 44 in Section Computation of helical parameters) is the mid step orientation matrix between the two base-pairs that comprise the bond. $\mathbf{T}$ can be thought of as the average orientation of the two base-pairs. The torque on a base-pair around the kth axis is computed numerically using the central finite difference,

$$\tau_k = -\frac{\partial E_{\text{RBP}}}{\partial \theta_k} \approx -\frac{E_{\text{RBP}}(+\Delta\theta_k) - E_{\text{RBP}}(-\Delta\theta_k)}{2\Delta\theta}, \tag{7}$$

where $\partial\theta_k$ represents an infinitesimal rotation about the kth axis and $+\Delta\theta_k$ represents rotating the current base-pair about its kth axis by the small rotation $\Delta\theta = 0.00001\,\text{rad}$.

Model building & implementation of the chemically-specific model

The chemically-specific coarse-grained model resolves DNA at the base-pair level and proteins at the amino acid level. The DNA beads are modeled using an ellipsoidal particle that approximates the shape and mass of a base-pair. The ellipsoid is rigidly connected to two point particles each with charge -1, which approximates the phosphate backbone and major/minor grooves. The DNA ellipsoids are spatially defined by a position vector $\mathbf{r}$, and a unit quaternion $\underline{q}$ which encodes the orientation and can be concerted into an orthogonal rotation matrix $\mathbf{A}$, whose columns are the unit axis vectors of the ellipsoids frame of reference.

We begin by taking our reference nucleosome structure as the most populated structure from our previous all-atom bias-exchange molecular dynamics metadynamics simulations of a 211-bp nucleosome [1]. From there, we define the position and orientation of the DNA ellipsoids by using the software 3DNA [13], which determines the coordinates of the rigid base pairs that fit the atomistic structure. The two phosphate point particles act as virtual charge sites, and for computational reasons have a negligible but non-zero mass of 1×10^{-6} g/mol. The three RBP particles (the ellipsoid plus the two point charges) are combined together as a rigid body using `fix rigid/nve/small` from the `RIGID` package in LAMMPS. The individual particles within the rigid body each contribute to the resultant potential and forces acting on the combined body, which is treated as a single rigid body for the dynamics.

Using the same atomistic reference structure from our 211-bp nucleosome simulations, the protein beads are defined as point particles centered at the C_α of each atomistic amino acid. The identity of the amino acid type is preserved by defining a different particle type for each unique amino acid. Each amino acid was classified as either belonging to a globular domain or histone tail (Supplementary table 1). The sequence-dependent mass, charge, van de Waals radius and hydrophobicity values for each amino acid type are given in Supplementary Table 2, which are taken from Ref. [9]. The classification was performed by estimating the persistence of protein secondary structures from our 211-bp nucleosome simulations [1].

The histone tails are modeled as fully flexible polymer chains (i.e., no energetic penalty for bending) with a bond energy modeled as in Eq. 2, with stiffness constant $k = 20\,\text{kcal/mol/Å}^2$ and equilibrium bond length $r_0 = 3.5\,\text{Å}$. Bonds are defined between adjacent amino acids along the protein backbone. The globular domains of the histones are regions which exhibit small structural fluctuations, and are largely α-helical. Using the 211-bp nucleosome reference structure, a Gaussian elastic Network Model (GNM) [14] is created by connecting all globular domain amino acid pairs that are closer than 7.5 Å, again using Eq. 2 where $k = 20\,\text{kcal/mol/Å}^2$ and r_0 is the bond length in the reference structure.

We develop two versions of the model: (a) breathing (i.e., with nucleosomal DNA that are allowed to unwrap spontaneously) and (b) non-breathing (i.e., with nucleosomes that are constrained to remain fully wrapped). In practice, these two versions differ in how the DNA beads are bound to the histone protein core. In the breathing case, DNA ellipsoids and amino acid beads interact exclusively via the electrostatic (Eq. 4) and Lennard-Jones potentials (hydrophobicity potential with λ=1, Eq. 5) defined above—this leaves the DNA free to bind and unbind spontaneously due to thermal fluctuations (i.e., "breathe"), and slide around the nucleosome core. In contrast, non-breathing simulations further constrain nucleosomal DNA by permanently bonding it to the histone core using a GNM with the same 7.5 Å threshold and bond parameters provided above, preventing these nucleosomes from breathing and sliding, and hence, forcing them to remain fully wrapped.

To simulate the presence of an implicit solvent and allow sampling of the canonical ensemble, we use Langevin dynamics. We use the following LAMMPS fixes: `fix nve` and `fix langevin` (using the Gronbech-Jensen/Farago formulation [15]) for the amino acid particles, and `fix rigid/nve/small` and `fix langevin` with angular momentum for the DNA beads.

To create the initial 12-nucleosome chromatin structure, we replicate a single nucleosome and join these copies together following the DNA double-helix. Care must be taken to avoid steric clashes so we use short non-equilibrium MD runs to pull the two DNA end points on adjacent nucleosomes, in opposite directions before joining them; this creates the chromatin array in a linear conformation that resembles an extended 'beads-on-a-string' structure.

Equilibration of structures is achieved by slowing decreasing, in steps, the friction coefficient of the Langevin thermostat whilst increasing the timestep. Unless otherwise specified, all production runs are performed at 300 K with a Langevin damping period of 100000 fs and a timestep of 40 fs; these values are the largest that ensure simulation stability. It is important to note that the timescales in these coarse-grained simulations are not directly comparable to timescales in atomistic simulations. The units of femtoseconds here are implemented to achieve compatibility with the units of the potentials.

Chemically-specific model parameters: Hydrophobic interactions

The parameters for all amino acid pair interactions are taken from Ref. [9], which provide the Kim-Hummer model parameter set A for interactions involving globular domains, and the parameter set D for interactions involving only histone tails. The mass, σ, and charge are given in Supplementary Table 2. Cross terms for σ, for use in Eq. 5, are calculated using the combination rule $\sigma = (\sigma_i + \sigma_j)/2$, where σ_i and σ_j are the individual σs for the two amino acids involved in the interaction. The cutoff distance is set at $r_c = 3\sigma$. The values of ϵ and λ for each amino acid pair can be found in the Supplementary Information of Ref. [9].

Due to the dominance of the electrostatic DNA self-repulsion at the salt conditions we explore in this work, we approximate the DNA–DNA pairwise hydrophobic interaction as zero: $\sigma_{\text{DNA–DNA}} = 0$, $\lambda_{\text{DNA–DNA}} = 1$, $\epsilon_{\text{DNA-DNA}} = 0$, with mass and charge as given in Supplementary Table 3.

To parameterize the hydrophobic interaction between DNA and the different amino acids—i.e., the parameters for the potential $E_{\text{Hydrophobic}}$ between DNA and amino acids—we fit these parameters to optimize the DNA ellipsoid–amino-acid and the DNA phosphate–amino acid radial pair-wise distance distribution functions (RDF) of the coarse-grained simulations to match that computed from our 211-bp all-atom simulations of single nucleosomes. We compute the RDF as:

$$g(r) = \frac{n_r}{4\pi r^2 \Delta r \rho}, \tag{8}$$

where n_r is the number of particles found at distance r in a spherical shell of thickness Δr. To find n_r we construct a histogram of all pair distances, where n_r is the bin height and Δr the bin width. ρ is the average density of the system. The fitted values are given in Supplementary Table 4.

Chemically-specific model parameters: Electrostatic interactions

We set the relative permittivity as that for water $\epsilon_r = 80$, to model the low concentration of monovalent ions within cells. The inverse Debye length κ, is varied with the monovalent salt concentration c (measured in units of mol/L) according to:

$$\kappa^{-1} = \lambda_D = \sqrt{\frac{\epsilon_0 \epsilon_r k_B T}{2 \times 10^3 N_A e^2 c}}; \tag{9}$$

where k_B is the Boltzmann constant, T is the temperature, N_A is the Avogadro constant and e is the elementary charge. We set the cutoff distance, $r_c = 3.5\lambda_D$.

Minimal Model

Minimal model potential energy function

The total potential energy of the minimal chromatin model is

$$E = E_{\text{LJ}} + E_{\text{Bonds}} + E_{\text{anisotropic}}. \tag{10}$$

E_{LJ} is a Lennard-Jones interaction

$$E_{\text{LJ}} = \sum_i \sum_{j<i} \begin{cases} 4\epsilon \left[\left(\frac{\sigma}{r}\right)^{12} - \left(\frac{\sigma}{r}\right)^6 \right], & r \leq r_c \\ 0, & r_c < r, \end{cases} \tag{11}$$

where r is the distance between the pair of interacting particles, ϵ, σ and r_c depend of the type of interacting particle.

E_{Bonds} is similar to the RBP potential of the chemically-specific model, indeed it has the same functional form,

$$E_{\text{Bonds}} = \sum \frac{1}{2} \Delta \boldsymbol{\phi}^{\intercal} \mathbf{K} \Delta \boldsymbol{\phi}. \tag{12}$$

This RBP-like potential is instead parameterized to model DNA at a resolution of 1 bead per 5 base pairs. At this resolution the effects of DNA sequence average out, therefore all DNA bonds have the same values for $\mathbf{K}$ and $\boldsymbol{\phi}_0$.

The anisotropic term is a pairwise potential that depends on the relative orientations and shape of the interacting pair of ellipsoidal particles.

$$E_{\text{anisotropic}} = \sum_i \sum_{j<i} U_r(\mathbf{A}_i, \mathbf{A}_j, \mathbf{r}) \eta(\mathbf{A}_i, \mathbf{A}_j, \mathbf{r}) \chi(\mathbf{A}_i, \mathbf{A}_j, \mathbf{r}), \tag{13}$$

where $\mathbf{A}_i$ and $\mathbf{A}_j$ are the orientation matrices of particles i and j with center to center separation vector $\mathbf{r}$. This potential is a modified version of the well-know Gay–Berne potential [16] where we have replaced the Lennard-Jones like term with a cosine-squared term [17]. This allows for greater control over the depth and range of the potential. The η and χ terms are unchanged from the original version in LAMMPS [18].

$$U_r = \begin{cases} -\epsilon & h < 0, \\ -\epsilon\cos\left(\frac{\pi h}{2r_c}\right)^2 & 0 \le h < r_c, \\ 0 & r_c \le h. \end{cases} \tag{14}$$

$$h = r - \sigma, \tag{15}$$

$$r = |\mathbf{r}|, \tag{16}$$

$$\sigma = \left[\frac{1}{2}\hat{\mathbf{r}}^{\intercal}\mathbf{G}^{-1}\hat{\mathbf{r}}\right]^{-1/2}, \tag{17}$$

$$\mathbf{G} = \mathbf{G}_i + \mathbf{G}_j = \mathbf{A}_i^{\intercal}\mathbf{S}_i^2\mathbf{A}_i + \mathbf{A}_j^{\intercal}\mathbf{S}_j^2\mathbf{A}_j, \tag{18}$$

where $\mathbf{S}_i = \mathrm{diag}(a_i, b_i, c_i)$ is the shape matrix of particle i given by the ellipsoid radii and r_c is the cutoff distance (numerical value is potential specific). The η and χ terms contain the Gay-Berne parameters μ,ν, and the relative energy matrices $\mathbf{E}_i = \mathrm{diag}(\epsilon_{i,x}^{-1/\mu}, \epsilon_{i,y}^{-1/\mu}, \epsilon_{i,z}^{-1/\mu})$ where $\epsilon_{i,k}$ is the depth of the potential well in the direction of the ith ellipsoids k^{th} axis. The anisotropic term only acts between the core particle and the DNA as it simulates DNA binding around the histone core.

The resulting potential of E_{LJ} and $E_{\text{anisotropic}}$ acting on DNA beads due to the core bead is graphed in Supplementary Figure 2.

A summary of all minimal model parameters is given in supplementary Table 10.

Anisotropic potential forces and torques

The forces and torques for the anisotropic potential are modified from the Gay–Berne implementation in LAMMPS [18, 19]. The force is

$$\mathbf{f} = -\eta\left(U_r\frac{\partial\chi}{\partial\mathbf{r}} + \chi\frac{\partial U_r}{\partial\mathbf{r}}\right), \tag{19}$$

where

$$\frac{U_r}{\partial\mathbf{r}} = \frac{\partial U_{CS}}{\partial r}\hat{\mathbf{r}} + r^{-2}\frac{\partial U_{CS}}{\partial\varphi}\left[\boldsymbol{\kappa} - (\boldsymbol{\kappa}^{\intercal}\hat{\mathbf{r}})\hat{\mathbf{r}}\right], \tag{20}$$

$$\frac{\partial U_{CS}}{\partial\varphi} = \frac{\sigma^3}{2}\frac{U_{CS}}{\partial r}, \tag{21}$$

$$\frac{\partial U_{CS}}{\partial r} = \begin{cases} 0, & h < 0, \\ \frac{\pi\epsilon}{2r_c}\sin\left(\frac{\pi h}{r_c}\right) & 0 \le h < r_c, \\ 0, & r_c \le h, \end{cases} \tag{22}$$

$$\boldsymbol{\kappa} = \mathbf{G}^{-1}\mathbf{r}. \tag{23}$$

$\partial\chi/\partial\mathbf{r}$ is the same as in [18]. The torque on particle i is given by

$$\boldsymbol{\tau}_i = U_r\eta\frac{\partial\chi}{\partial\mathbf{q}_i} + \chi\left(U_r\frac{\partial\eta}{\partial\mathbf{q}_i} + \eta\frac{\partial U_r}{\partial\mathbf{q}_i}\right), \tag{24}$$

where

$$\frac{\partial U_r}{\partial\mathbf{q_i}} = \mathbf{A}_i\left(-\boldsymbol{\kappa}^\intercal\mathbf{G}_i \times \left[-r^{-2}\frac{\partial U_{CS}}{\partial\varphi}\boldsymbol{\kappa}\right]\right), \tag{25}$$

and $\partial\chi/\partial\mathbf{q}_i$ and $\partial\eta/\partial\mathbf{q}_i$ are the same as in [18].

Model building & implementation of the minimal model

The minimal model uses two types of ellipsoidal particles. One represents the nucleosome core and is disk shaped (referred to as a core bead), the other type represents 5 DNA base pairs (referred to as a DNA bead) and is actually spherical but still uses the LAMMPS ellipsoidal type for computational compatibility. The radii of the core beads is $28 \times 28 \times 20$ Å and the radii of the DNA beads is $12 \times 12 \times 12$ Å, these have been optimized as part of the fitting procedure. The beads are further categorized into linker and nucleosomal DNA, the nucleosomal DNA is fixed rigidly together with the core bead it is wrapped around to form a rigid nucleosome. The linker DNA beads are left free, bonded in sequence by the minimal RBP potential. The reference structures for creating the minimal model structures are the equilibrium structures from the chemically-specific model simulations (Supplementary Figure 3). The minimal coarse-grained DNA beads are positioned by grouping base-pairs in sets of 5 and taking the average position and orientation. The minimal coarse-grained core beads are positioned by taking the center of mass of the nucleosome core globular domain. The orientation is set by using specific amino acid beads to consistently construct x,y,z orientation axis vectors of each nucleosome.

The difference between the non-breathing and breathing in the minimal coarse-grained model is only the initial structures that are used, both have the nucleosomal DNA rigidly fixed to the nucleosome core. For the breathing structures the effects of DNA unbinding/sliding is fixed into the structure from the equilibrated chemically-specific coarse-grained structure that was used as the reference. This means that although during a minimal coarse-grained simulation the DNA cannot further dynamically breathe, it is in configurations that represent the thermodynamic fluctuations that occur due to DNA breathing.

Once again Langevin dynamics is used, but for the minimal coarse-grained model the damping time constant is 5 ns and the timestep is 500 fs, the temperature is still 300 K. The particle masses are kept consistent with the chemically-specific model with the DNA beads having a mass of 3250 g/mol and the core beads having a mass of 100,000 g/mol.

Fitting of the minimal model parameters from chemically-specific model simulations

We fit the parameters of the minimal model such that it approximates the salt dependent behavior of the chemically-specific model.

To obtain initial values for Eq. 11 we first computed the inter-nucleosome potentials of mean force (PMFs) using the chemically-specific model for high and low salt, these are shown in Supplementary Figure 4 a1 and a2. The method used to calculate these is described in Section Details of the chemically-specific model nucleosome-nucleosome potential of mean force simulations. We then performed a similar calculation using the minimal model. Due to the fact that the minimal nucleosomes are completely rigid, umbrella sampling is not needed, instead we simply compute the potential energy as a function of the inter-nucleosome distance. We optimize the values of σ and ϵ such that the minimal model best approximates the shapes of chemically-specific inter-nucleosome PMFs. Armed with these initial guesses of σ and ϵ, which are the end points of the salt range we wish to model, we proceed to find an adequate interpolation to model the salt dependent behavior. To do this we use the radius of gyration of non-breathing 12-nucleosome chromatin as the observable to compare between the minimal model and the chemically specific model. Using a combination of manual adjustment and grid search techniques we obtain the optimal parameters (in Supplementary Table 5) that give the R_g values in Supplementary Figure 4 b2; yielding good agreement with the chemically specific model.

Moving on to the breathing model and comparing the radii of gyration between the minimal model and the chemically-specific model (red and green lines in Supplementary Figure 4 b2), we find a significant difference in the behavior at higher salt values. This is due to the unwrapped DNA not having strong enough interactions to the exposed nucleosome cores. To account for this we developed the anisotropic potential which provides a strong short range attractive binding potential around the nucleosome core where the DNA is located in an archetypal nucleosome.

To fit the parameters of the aniostropic potential we first use our knowledge of the shape of the nucleosome core and the region where the DNA binds. This allows us to set the values of the ellipsoid shape matrices $\mathbf{S}_{\text{core}}$ and $\mathbf{S}_{\text{DNA}}$, and the anisotropic energy matrices $\mathbf{E}_{\text{core}}$ and $\mathbf{E}_{\text{DNA}}$. Specifically $\mathbf{E}_{\text{core}}$ is constructed such that there is no attraction at the z-poles of the ellipsoid. We then ensure that it is short ranged enough to have no effect on the non-breathing minimal model. That is, the potential well is completely covered by the bound nucleosomal DNA. This gives us the value of cutoff r_c, and the correspondence of the green and blue points in Supplementary Figure 4b1 demonstrates the value ensures the anisotropic potential has no effect on the non-breathing minimal model. To fit the remaining parameter ϵ, the depth of the potential, we compute the PMF of nucleosome unwrapping for a minimal model nucleosome where the DNA is completely free from the core and only the pairwise interactions keep it bound. We perform a grid search parameter sweep and chose the value of 6 kcal/mol which gives the PMF that best approximates the chemically-specific PMF in the low extension regime. The resulting PMF is shown in Supplementary Figure 4c.

Finally, the minimal RBP-like helical parameters are optimized by directly fitting to chemically-specific coarse-grained simulations of 200bp DNA strands. 10 simulations of random DNA sequences are performed for 10 million timesteps. The chemically-specific coarse-grained trajectories are then mapped into the minimal representation. For each timestep and each bond, the helical parameters between the minimal ellipsoids are computed. We now have histograms of the equilibrium distributions for each of the six helical parameters. We observe that shift, slide, tilt, and roll are centered about roughly zero, so we set these equilibrium values to zero. Rise and twist are set to the calculated mean values. The stiffness matrix is constructed by computing the variance of each helical parameter.

$$\mathbf{K} = k_B T \begin{pmatrix} k_{\text{shift-slide}} & 0 & 0 & 0 & 0 & 0 \\ 0 & k_{\text{shift-slide}} & 0 & 0 & 0 & 0 \\ 0 & 0 & k_{\text{rise}} & 0 & 0 & 0 \\ 0 & 0 & 0 & k_{\text{tilt-roll}} & 0 & 0 \\ 0 & 0 & 0 & 0 & k_{\text{tilt-roll}} & 0 \\ 0 & 0 & 0 & 0 & 0 & k_{\text{twist}} \end{pmatrix}, \tag{26}$$

$$k_{\text{shift-slide}} = [(\text{Var(shift)} + \text{Var(slide)})/2]^{-1}, \tag{27}$$
$$k_{\text{rise}} = \text{Var(rise)}^{-1}, \tag{28}$$
$$k_{\text{tilt-roll}} = [(\text{Var(tilt)} + \text{Var(roll)})/2]^{-1}, \tag{29}$$
$$k_{\text{twist}} = \text{Var(twist)}^{-1}, \tag{30}$$

where Var represents taking the variance. Note that here, unlike the original base-pair resolution RBP model, we neglect the off diagonal covariance terms. Furthermore, shift and slide are set equal, and tilt and roll are set equal. At this coarse-grained level we are unable to resolve the DNA major/minor grooves so the approximation of symmetry around the z-axis is acceptable. These approximations are an important step as it allows us to redefine the equilibrium value of twist to be zero and remap the minimal DNA in the structures by rotating each DNA bead by:

$$\text{rotation correction} = (\text{position along sequence counting from zero}) \times -(\text{initial twist value}) \tag{31}$$

This is needed because the initial twist value of 167° causes numerical instabilities in the current computational implementation of the RBP model due to the angles wrapping around from 180° to -180°. We confirm that the DNA persistence length of the minimal model is the similar to that of the chemically-specific model.

Parameters of the minimal model potentials

The Lennard-Jones interaction parameters are in Supplementary Table 5, some are dependent on the salt concentration c.

The RBP-like bonds are have the following parameters:

$$\boldsymbol{\phi}_0 = (0, 0, 16.4, 0, 0, 0). \tag{32}$$

$$\mathbf{K} = \begin{pmatrix} 0.198 & 0 & 0 & 0 & 0 & 0 \\ 0 & 0.198 & 0 & 0 & 0 & 0 \\ 0 & 0 & 1.148 & 0 & 0 & 0 \\ 0 & 0 & 0 & 0.00448 & 0 & 0 \\ 0 & 0 & 0 & 0 & 0.00448 & 0 \\ 0 & 0 & 0 & 0 & 0 & 0.0027 \end{pmatrix} \quad (33)$$

where the units are Å for distances, degrees for angles, kcal/mol/Å^2 for spatial stiffness matrix elements, and kcal/mol/degrees^2 for angle stiffness matrix elements.

The parameters for the anisotropic potential are in Supplementary Table 7.

Simulation protocols

Debye-length replica exchange simulation method

We implement our Debye length Hamiltonian Replica Exchange Molecular Dynamics (HREMD) method via a modified version of the existing LAMMPS parallel tempering command from the `REPLICA` package.

Simultaneous simulations are performed on different replicas of the system, with the implicit salt concentration varying (by changing the Debye length) between replicas. Each time a Hamiltonian exchange between two replicas is attempted, the potential energies of the replicas are calculated as if they have swapped Debye lengths. The exchange probability is then determined using the Metropolis criteria,

$$P(i \leftrightarrow i+1) = \min\left(1, \exp\left[\frac{1}{k_B T}\left(U_{\lambda_D^i}(\boldsymbol{x}^i) - U_{\lambda_D^i}(\boldsymbol{x}^{i+1}) + U_{\lambda_D^{i+1}}(\boldsymbol{x}^{i+1}) - U_{\lambda_D^{i+1}}(\boldsymbol{x}^i)\right)\right]\right), \quad (34)$$

where $\boldsymbol{x}^i$ is the chromatin coordinates of the ith replica and $U_{\lambda_D^i}$ the potential energy function at Debye length λ_D^i—the original Debye length of the ith replica. Unlike temperature replica exchange, HREMD requires the recalculation of the potential energy. However, here only the re-computation of $E_{\text{Electrostatic}}$ is necessary, and as a replica exchange attempt is only made infrequently this does not significantly degrade performance.

Details of chemically-specific model 12-nucleosome chromatin simulations

HREMD simulations with 16 replicas with Debye lengths ranging from 8–15 Å (see Supplementary Table 8) are performed for 12-nucleosome chromatin arrays with NRLs of 165 bp and 195 bp in both the breathing and non-breathing cases. The simulations were run for $\sim$100 million timesteps and a set of exchanges were attempted every 10,000 timesteps. Each set of exchanges either attempts to exchange replicas {1-2,3-4...15-16} or {2-3,4-5...14-15}, with each set picked with a 50% probability. Coordinate snapshots were recorded to the trajectories every 100,000 timesteps. The last 50 million timesteps were used for analysis.

For qualitative assessment of chromatin dynamics an equilibrium structure was taken from the 0.1 mol/L simulations for both the breathing and non-breathing simulations, and run using standard MD (non replica exchange) for $\sim$40 million timesteps. They were used for the generation of the $k = 2$ interaction time series plotted in main text Figure 5.

Details of the chemically-specific model nucleosome unwrapping potential of mean force simulations

The force–extension simulations of mononucleosomes were performed following the umbrella sampling procedure proposed by Lequieu et al. [20] but with a tension of zero. The collective variable is the DNA extension which is the distance between the first and last base-pair. We implement the umbrealla sampling simulations using the COLVARS library in LAMMPS [21] (version 2019-08-05). Starting from an equilibrium structure with an extension of 25 Å, initial configurations for the windows were prepared via constant velocity steered MD (SMD) until the extension was at 750 Å. A spring constant of 0.01 kcal/mol/Å^2 was used with a pulling velocity of 9.0×10^{-6} Å/fs, giving a total pulling time of 100 ns. The extension range was split into 50 equally spaced windows. Each window was run with a fixed harmonic biasing potential at the corresponding extension with a spring constant of 0.025 kcal/mol/Å^2 for 100 ns. These values were chosen by assessing histogram overlap and checking that the calculated potential of mean forces (PMF) were the same on longer timescales. The entire procedure was repeated 5 times and the aggregate data was used for computing the PMF via the Weighted Histogram Analysis Method (WHAM) [22]. The same procedure was done for all nucleosome configurations and environments.

Details of the chemically-specific model nucleosome-nucleosome potential of mean force simulations

Due to the disk like shape of nucleosomes the interaction is divided into three orientation dependent configurations: face-face, face-side, and side-side. A PMF is constructed for each configuration using umbrella sampling as follows. An initial single nucleosome structure is equilibrated, it is then replicated and each copy is positioned such that the center to center distance is 200 Å. The nucleosomes are rotated into the correct orientations and held fixed using COLVARS' [21] `angleOrient` collective variable with a harmonic restraint of strength 1 kcal/mol/degrees^2 with center 0°. We use LAMMPS `fix spring tether` to restrain the nucleosomes in the simulation box x and y directions to lie along the simulation box z axis with harmonic restraints of strength 1 kcal/mol/Å^2. Motion in the z direction is unaffected by this fix. The final collective variable is distance between the nucleosome centers, R.

To prepare initial configurations for the windows, R is varied using a SMD protocol from its initial value of 200 Å to its final value of 10 Å over 1 million timesteps with a force constant of 0.1 kcal/mol. The range of R was split into 39 equally spaced windows, from 10 to 200, and each window was run for 10 million timesteps at the corresponding value of R with a force constant of 0.05 kcal/mol/Å^2. The orientational collective variables are kept with the same restraints as in the setup stage. Finally the PMFs were computed from the window trajectories using WHAM [22].

Estimation of radius of gyration from simulations with our minimal model

For the radius of gyration simulations plotted in Supplementary Figure 4 we first obtain 50 random initial 12-nucleosome structures by generating them (as described in Section Model building & implementation of the minimal model) from randomly selected, equilibriated, structures from the chemically-specific model HREMD simulations at the corresponding salt. We then run the 50 minimal model 12-nucleosome structures for 500,000 timesteps. These 50 repeats are done for each plotted salt value.

Details of the minimal model nucleosome unwrapping potential of mean force simulations

To calculate the PMF plotted in Supplementary Figure 4 we once again use the COLVARS library and follow a very similar procedure to Section Details of the chemically-specific model nucleosome unwrapping potential of mean force simulations. The distance between the first and last DNA bead R, is varied from 100 Å to 700 Å in 60 steps, with each step lasting 10,000 timesteps. The time series of R is split into the corresponding 60 windows and WHAM [22] is used to calculate the PMF.

Direct coexistence simulations

In order to compute the phase diagram of 12-nucleosome chromatin we employ the direct coexistence method [23–25] using 125 independent 12-nucleosome chromatin arrays with 165-bp NRL at different conditions. The chromatin array initial structures are obtained by generating them from randomly selected, equilibrated, structures from the chemically-specific model HREMD simulations at the corresponding salt.

In a direct coexistence simulation one places both phases; i.e., the dilute liquid and condensed liquid phase in the same box. The simulation is performed until they reach equilibrium at their coexistence densities. Once equilibrium is reached to measure the density of coexistence we compute an average density profile along the long side of the simulation box with the center of mass fixed. The density profiles for our simulations are shown in Supplementary Figure 5. Once equilibrated the simulations were run for approximately 100 million timesteps and coordinate snapshots were recorded every 10,000 timesteps. This is greater than the minimal model R_g correlation time of 2 ns (see Section Timescale difference between chemically-specific and minimal model) which is equal to 4000 timesteps (1 timestep = 500 fs). The simulation box dimensions were (1200Å × 1200Å × 5000Å).

We estimate the critical salt concentration c_c by fitting the density difference between the coexisting low-density $\rho_l(c)$ and high-density $\rho_h(c)$ phases to the expression [26],

$$(\rho_h(c) - \rho_l(c))^{3.06} = d\left(1 - \frac{c}{c_c}\right), \tag{35}$$

where d is a fitting parameter. The critical density ρ_c is estimated using the law of rectilinear diameter,

$$(\rho_l(c) + \rho_h(c))/2 = \rho_c + s\,(c_c - c), \tag{36}$$

where s is a fitting parameter.

Additional Algorithms

Quaternion to rotation matrix

The unit quaternion

$$\underline{q} = (q_w, q_x, q_y, q_z), \tag{37}$$

$$|\underline{q}| = \sqrt{q_w^2 + q_x^2 + q_y^2 + q_z^2} = 1, \tag{38}$$

can be converted into an orthogonal rotation matrix:

$$\mathbf{A} = \begin{pmatrix} q_w^2 + q_x^2 - q_y^2 - q_z^2 & 2(q_x q_y - q_w q_z) & 2(q_x q_z + q_w q_y \\ 2(q_x q_y + q_w q_z) & q_w^2 - q_x^2 + q_y^2 - q_z^2 & 2(q_y q_z - q_w q_x) \\ 2(q_x q_z - q_w q_y) & 2(q_y q_z + q_w q_x) & q_w^2 - q_x^2 - q_y^2 + q_z^2 \end{pmatrix}. \tag{39}$$

Computation of helical parameters

We compute the helical parameters of the RBP model using the SCHNAaP procedure [8]. The two base-pairs (DNA ellipsoids) have positions and orientation quaternions $\mathbf{r}_1, \underline{q}_1$ and $\mathbf{r}_2, \underline{q}_2$ respectively. The following method computes the helical parameters $\boldsymbol{\phi} = (D_x, D_y, D_z, \tau, \rho, \Omega)$ that describe their relative orientation:

1. Convert quaternions $\underline{q}_1$ and $\underline{q}_2$ to matrices $\mathbf{T}_1$ and $\mathbf{T}_2$ whose columns are the $\hat{\mathbf{x}}, \hat{\mathbf{y}}, \hat{\mathbf{z}}$ axis direction vectors.
2. Calculate the roll-tilt angle Γ:
$$\Gamma = \cos^{-1}(\hat{\mathbf{z}}_1 \cdot \hat{\mathbf{z}}_2). \tag{40}$$
3. Calculate the roll-tilt axis $\mathbf{rt}$:
$$\mathbf{rt} = \hat{\mathbf{z}}_1 \times \hat{\mathbf{z}}_2. \tag{41}$$
4. Rotate base-pair 1 and 2 about $\mathbf{rt}$ by $+\Gamma/2$ and $-\Gamma/2$ respectively:
$$\mathbf{T}_1' = \mathbf{R}(\mathbf{rt}, +\Gamma/2)\mathbf{T}_1, \tag{42}$$
$$\mathbf{T}_2' = \mathbf{R}(\mathbf{rt}, -\Gamma/2)\mathbf{T}_2, \tag{43}$$
where $\mathbf{R}(\mathbf{a}, \theta)$ is an orthogonal matrix that describes a rotation of θ about axis $\mathbf{a}$.
5. The mid-step matrix $\mathbf{T}$ is the mean of the rotated matrices:
$$\mathbf{T} = \frac{1}{2}(\mathbf{T}_1' + \mathbf{T}_2'). \tag{44}$$
6. Twist Ω is the angle between the transformed y axis ($\hat{\mathbf{y}}_1'$ is the second column of $\mathbf{T}_1'$):
$$\Omega = \cos^{-1}(\hat{\mathbf{y}}_1' \cdot \hat{\mathbf{y}}_2'), \tag{45}$$
7. Calculate ϕ the angle between the roll-tilt axis and the mid-step y-axis ($\hat{\mathbf{y}}_{ms}$ is the second column of $\mathbf{T}$):
$$\phi = \cos^{-1}(\hat{\mathbf{rt}} \cdot \hat{\mathbf{y}}_{ms}). \tag{46}$$
8. Roll ρ and tilt τ are given by:
$$\rho = \Gamma \cos(\phi), \tag{47}$$
$$\tau = \Gamma \sin(\phi). \tag{48}$$
9. Shift Dx, slide Dy, and rise Dz are calculated as:
$$(Dx, Dy, Dz)^{\intercal} = \mathbf{T}(\mathbf{r}_2 - \mathbf{r}_1)^{\intercal}. \tag{49}$$

Analysis of the chemically-specific model simulations

Orientation dependent nucleosome-nucleosome interactions

The relative orientation of two nucleosomes can be categorized into three states: face-face (ff), face-side (fs), and side-side (ss) as illustrated in Supplementary Figure 6. To characterize these, we compute the nucleosome orientation matrices—the columns of which are the orthogonal unit axis vectors of the nucleosome. The center of a nucleosome is defined as the center of mass of the globular domain beads. The x axis passes through the nucleosome dyad and the z axis points perpendicularly out of the nucleosome "face" as shown in Supplementary Figure 6.

Below, we explain the procedure used to categorize the relative orientation of the nucleosomes. We define the angles $\{\alpha, \beta_i, \beta_j\}$ as

$$\hat{\mathbf{z}}_i \cdot \hat{\mathbf{z}}_j = \cos\alpha, \tag{50}$$

$$\hat{\mathbf{z}}_i \cdot \hat{\mathbf{r}} = \cos\beta_i, \tag{51}$$

$$\hat{\mathbf{z}}_j \cdot \hat{\mathbf{r}} = \cos\beta_j, \tag{52}$$

where $\hat{\mathbf{r}}$ is the unit vector pointing from the center of the ith nucleosome to the center of the jth nucleosome, and $\hat{\mathbf{z}}_i$ and $\hat{\mathbf{z}}_j$ are the unit z-axis vectors of ith and j^{th} nucleosomes respectively. We then use following procedure:

if $\alpha < 45°$ or $\alpha > 135°$:
  if $\beta_i < 45°$ or $\beta_i > 135°$ or $\beta_j < 45°$ or $\beta_j > 135°$:
    Face-face
  else: (53)
    Side-side
else:
  Face-side

We then construct three interaction matrices M^{μ}_{ij} between the ith and j^{th} nucleosomes, one for each relative orientation $\mu = \{\text{ff}, \text{fs}, \text{ss}\}$:

$$M^{\mu}_{ij} = \frac{1}{N_t} \sum_t C^{\mu}_{ij}(t), \tag{54}$$

$$C^{\mu}_{ij}(t) = \begin{cases} 1, & \text{if nucleosomes i and j are in contact, and have a type } \mu \text{ relative orientation,} \\ 0, & \text{otherwise,} \end{cases} \tag{55}$$

where t is the timestep. The sum is taken over all N_t snapshots used in the analysis. Two nucleosomes are defined to be in "contact" when the center to center distance between them is $< 110\,\text{Å}$. The interaction matrices can then be projected onto a 1D map to describe the relative intensity of interactions between nucleosomes separated by $(k-1)$ neighbors,

$$I^{\mu}(k) = \frac{1}{N_n} \sum_i M^{\mu}_{i,i+k}. \tag{56}$$

Sedimentation coefficient

The sedimentation coefficient is computed using the HullRad method [27]:

$$s = 10^8 \left(\frac{M - M\bar{v}\rho_{20,w}}{N_A 6\pi\eta_0 R_T} \right), \tag{57}$$

where M is the total molar mass, $\bar{v}$ is the total partial specific volume, $\rho_{20,w}$ is the density of water at 20C, and η_0 is viscosity of water at 20C.

$$M = \sum_i m_i, \tag{58}$$

$$\bar{v} = \frac{1}{M} \sum_i m_i v_i, \tag{59}$$

where m_i and v_i are the molar masses and specific volumes of the individual beads. R_T is the translational hydrodynamic radius which is computed via generating the convex-hull of the molecule. Full details of this calculation can be found in [27].

Amount of unwrapped DNA

For the simulations of breathing chromatin it becomes slightly difficult to define which DNA beads are nucleosomal and which are linker. This is because at the higher salt values the dense chromatin structures have DNA in contact with the nucleosome core proteins that is not part of that nucleosome, so simply computing the protein-DNA contacts will not work. To overcome this we developed the follow procedure: first we record which protein beads are bonded to the DNA in simulations of non-breathing nucleosomes, these protein beads are located circularly around the nucleosome in the locations where the DNA is typically wrapped. Then, for each frame in the breathing trajectory, we compute the contacts between the DNA and the aforementioned protein beads. For each nucleosome we now have a list of bound DNA beads. We then compute the median DNA bead in terms of index along the DNA sequence. This is approximately the center bead of that nucleosome's DNA. We then look forwards and backwards along the DNA sequence, within the range of maximum and minimum indices of the bound DNA beads, and unless a large continuous region of unbound DNA (>100bp) is found, all the DNA between the maximum and minimum limits is added. Each nucleosome now has a contiguous section of nucleosomal DNA assigned to it. Finally the list of nucleosomal DNA is checked for overlaps and any are removed to ensure that each DNA bead can only be a member of one nucleosome. The average amount of unwrapped DNA per nucleosome is then computed as:

$$N_{\text{unwrapped}} = (147 N_n - N_{\text{nucleosomal DNA}})/N_n, \tag{60}$$

where N_n is the number of nucleosomes, $N_{\text{nucleosmal DNA}}$ is the total number of nucleosomal DNA beads, and 147 is the typical number of base pairs of DNA wrapped round one nucleosome.

Nucleosome valency

Nucleosome valency V, is defined as the average number of other nucleosomes a nucleosome is in contact with, as defined in Section Orientation dependent nucleosome-nucleosome interactions .

$$V = \frac{1}{N_t N_n} \sum_t^{N_t} \sum_{i,j}^{N_n} C_{ij}(t), \tag{61}$$

$$C_{ij}(t) = \begin{cases} 1, & \text{if nucleosomes i and j are in contact,} \\ 0, & \text{otherwise.} \end{cases} \tag{62}$$

Nucleosome–nucleosome contacts

To calculate the inter-nucleosome molecular contacts we use a similar procedure to the nucleosome-nucleosome interactions but at bead level rather than nucleosome level. For each timestep in the simulation trajectory the total contact matrix is computed for all beads.

$$M_{ij} = \begin{cases} 1, & \text{if beads i and j are in contact} \\ 0, & \text{otherwise,} \end{cases} \tag{63}$$

where "in contact" here is true when the distance between beads i and j is less than $((\sigma_i + \sigma_j)/2 + 1\text{Å})$. For each bead three sums are performed. One each counting up the contacts with: DNA, Histone tail, and Globular domain beads. Importantly the contacts are only counted if they are located in different nucleosomes. For the breathing DNA this is non-trivial. To proceed we defined nucleosomal DNA using the same method as in Section Amount of unwrapped DNA. The remaining linker DNA is then assigned to the nucleosome it is closest to (in term of DNA sequence, not spatial distance). This process enables the contacts to be computed for interactions between different nucleosomes that would otherwise be dominated by the intra-nucleosome contacts.

$$C^{\text{x}}(i) = \sum_{j \text{ in x and in different nucleosome to i}} M_{ij}, \quad \text{x} = \{\text{DNA}, \text{Histone tails}, \text{Globular domain}\}. \tag{64}$$

$C^{\mathrm{x}}(i)$ is then averaged over all nucleosomes and all timesteps in the trajectory, and normalized by its maximum value C_{MAX}. The values of $C^{\mathrm{x}}(i)$ are plotted in Figure 4a and 4c. To generate the visualizations in Figure 4b and 4d each bead is given a RGB color according to:

$$\mathrm{color}_i = (\mathrm{red}, \mathrm{green}, \mathrm{blue}) = 255 \frac{\log_{10}\left(C_{\mathrm{MAX}} \times (C^{\mathrm{DNA}}(i), C^{\mathrm{Globular\ domain}}(i), C^{\mathrm{Histone\ tails}}(i))\right)}{\log_{10}(C_{\mathrm{MAX}})}. \tag{65}$$

Where RBG values are integers in the range 0-255.

Analysis of minimal simulations

Radius of gyration

To enable comparison with the chemically-specific model the radius of gyration is computed using only the core beads:

$$R_g = \sqrt{\frac{1}{N}\left\langle \sum_i^N (r_i - r_{\mathrm{mean}})^2 \right\rangle}. \tag{66}$$

Estimation of liquid-network connectivity

We define the connectivity as the mean number of connections per chromatin array in the high-density phase multiplied by the density of high-density phase, which gives the connectivity as the number of inter-chromatin bonds per unit volume. The number of connections of a chromatin array is defined as the number of distinct chromatin arrays it is in contact with. Two nucleosomes are defined to be “in contact" if the nucleosome–nucleosome distance (i.e. any nucleosome in one chromatin array relative to any nucleosome in another chromatin array), is less than 110Å.

Supplementary Notes

Timescale difference between chemically-specific and minimal model

To approximate the difference in timescales going from the chemically-specific model to the minimal model we compute the autocorrelation function of the radius of gyration of a 12-nucleosome chromatin system at low salt conditions in both models. The radius of gyration is calculated from the center of masses of the nucleosomes in both models. The time series are plotted in Supplementary Figure 7a and b for the chemically-specific model and the minimal model respectively. We compute the autocorrelation function $C(\tau)$ using

$$C(\tau) = \frac{\langle (R_t - \bar{R})(R_{t+\tau} - \bar{R}) \rangle}{\mathrm{Var}(R)}, \tag{67}$$

where τ is the time lag, R_t are the values of the radius of gyration at timestep t, $\bar{R}$ is the mean radius of gyration, $\mathrm{Var}(R)$ is the variance, and the averages are taken over t. The auto-correlation functions are plotted in 7 c and d, the values of the correlation time t_c are approximated by reading of the graphs where the value of $C(\tau)$ reaches $1/e$.

Nucleosome formation

We investigate the ability of the chemically specific model to spontaneously form nucleosomes starting from configurations where the DNA is completely unwrapped and unbound from the histone core. These simulations are performed by setting up the structures, as pictured in the left panel of Supplementary Figure 8a, in a periodic simulation box. The box dimensions are approximately double the length of the fully extended DNA, the periodicity ensures the DNA and nucleosome will eventually come in to close enough contact so they can interact. The simulations are run until binding occurs and then run for a further 10 million timesteps to ensure the resulting structures are well equilibrated. 64 trials were run and categorized by visual inspection into the pie chart categories in Supplementary Figure 8a. We observe that a nucleosome is only correctly formed approximately one third of the time, the other resulting configurations include: a reversome, which is when the DNA is wrapped in a right-handed helix; “knotted" configurations where the DNA coiling is nucleosome-like but has DNA overlap; finally “wrong" where the DNA coiling is not nucleosome-like. This suggests additional processes are required to ensure the model always forms nucleosomes. Experimental literature shows that with completely relaxed DNA the formation of nucleosomes is significantly slowed, with positive supercoiled DNA nucleosomes do not form, and with negative supercoiled DNA the nucleosomes form instantaneously [28]. To investigate the response of the model to DNA

supercoiling we repeat the simulations with the addition of torsional restraints to the DNA strand which is setup with either negative or positive supercoiling. The supercoiling is achieved by either increasing (for positive) or decreasing (for negative) the twist angle between base-pairs by 10%. The torsional restraints fix the rotation of the end-two base-pair by the addition of strong additional forces such that they cannot rotate about their z-axis. Additionally we start the simulation with the DNA in contact with the histone core. Performing 64 repeats we observe that negative super-coiling always forms a nucleosome, consistent with the experimental literature, and positive supercoiling always forms the chirally inverted reversome (Supplementary Figure 8b). The reversome is a metastable state of a nucleosome that is observed in experiments [29–31].

Persistence length of DNA

To test our chemically-specific coarse-grained DNA model we compute the persistence length of DNA as a function of monovalent salt concentration (Supplementary Figure 9a) and DNA sequence (Supplementary Figure 9b). The simulations are performed using 300 bp long strands of isolated DNA. 10 repeats are done for each data point for a total simulation time of ∼100 million time steps. The persistence length P of polymer is the length at which correlations on the direction of the polymer tangent are lost. We use the following definition:

$$\langle \mathbf{n}(x_i) \cdot \mathbf{n}(x_j) \rangle = e^{-l/P}, \qquad (68)$$

$$l = |x_i - x_j|.$$

Where $\mathbf{n}(x_i)$ is the tangent vector of the polymer at location x_i—note that location here is in terms of contour distance with units of base-pairs (symbol bp). E.g., $x_1 = 1$ is the 1st base-pair and $x_{10} = 10$ is the 10th base-pair. l is the contour distance. To compute the value of P from our simulations we use the DNA ellipsoids' quaternion orientation to directly give the tangent vectors. The end 20 base-pairs are excluded from analysis. For each time step all pairs of $\mathbf{n}(x_i) \cdot \mathbf{n}(x_j)$ are computed and the average is taken over all timesteps for each l value. We then plot the scatter graph of l verses $\langle \mathbf{n}(x_i) \cdot \mathbf{n}(x_j) \rangle$ and fit an exponential curve to obtain P.

We see reasonable agreement with the experimental data for the salt dependence, the differences between our values and the experimental are of the same order as the differences between the the two experimental plots. This highlights the difficulty in accurate measurement of the persistence length of semi-flexible polymers with the existence of multiple techniques and alternative definitions to our Eq. 68 [32–34]. For the sequence dependent behavior our results follow the general trend where stiffness decreases going from left to right across the sequences. All but one data point is either in agreement with the experimental or the MD results. There are significant differences between all the different plots which can be explained by the aforementioned difficulty in accurate persistence length calculations.

Comparison with force-spectroscopy experiments of mononucleosomes

The force-extension curves in Supplementary Figure 10a are computed as the numerical derivative of the PMF curves in figure 2a of the main text. The rupture forces listed in Figure 2c are the values at the peaks, labeled as F_1 and F_2 in Supplementary Figure 10a, that occur at the transition states.

During the first regime (state 1), the force increases moderately as the DNA extends until it reaches a local maximum. The force then drops signaling the DNA-protein interactions being broken as the outer DNA turn unwraps. In the second regime (state 2), a similar behavior occurs with the force increasing significantly as the DNA continues to extend, and then reaching a second maximum and dropping again. This second drop in the force corresponds to the rupture of the DNA-protein interactions in the inner DNA turn. The third and final regime (stage 3) reflects the stretching of the fully unwrapped, but still histone-bound, nucleosomal DNA.

The forces required to unwrap the outer and inner DNA turns according to our model—e.g., ∼5 pN and ∼30 pN at 0.15 mol/L for the outer and inner DNA turn, respectively—are in accordance with the values from force spectroscopy experiments [35–37].

Force-extension simulations of oligonucleosomes

As further validation, we conducted force-extension simulations of 4-nucleosome chromatin arrays by performing constant velocity steered MD. The spring constant was 0.001 kcal/mol/Å^2 and the pulling velocity was 3.0×10^{-6} Å/fs. The initial structures were equilibrated for 100 ns and then the SMD procedure was run for 1000 ns. Values of the applied SMD force and current extension are stored at each timestep (Supplementary Figure 10b).

In Supplementary Figure 10b, we compare the force-extension response of a 4-nucleosome system using our breathing model and the non-breathing model. Our force-extension curves exhibit the typical saw-toothed pattern observed in optical tweezer experiments of chromatin [38]; that is, the force exhibits an abrupt drop accompanied

by a certain increase in the extension due to the partial unwrapping of individual nucleosomes. When we use the non-breathing model where the nucleosomal DNA remain permanently bound to the histone core the pattern disappears, and a much stiffer chromatin emerges.

Results for a longer NRL

Using our chemically-specific model, we performed additional simulations for 12-nucleosome arrays with uniform NRL of 195 bp (Supplementary Figure 11).

Supplementary Tables

Histone	Tail region residues
H3	1-40
H4	1-25
H2A	1-20, 114-128
H2B	1-25

Supplementary Table 1. Definition of histone tail regions. All other residues are classified as belonging to the central globular region.

Amino acid	Mass (g/mol)	σ (Å)	Charge (e)
ALA	71.08	5.04	0
ARG	156.2	6.56	1
ASN	114.1	5.68	0
ASP	115.1	5.58	-1
CYS	103.1	5.48	0
GLN	128.1	6.02	0
GLU	129.1	5.92	-1
GLY	57.05	4.50	0
HIS	137.1	6.08	0.5
ILE	113.2	6.18	0
LEU	113.2	6.18	0
LYS	128.2	6.36	1
MET	131.2	6.18	0
PHE	147.2	6.36	0
PRO	97.12	5.56	0
SER	87.08	5.18	0
THR	101.1	5.62	0
TRP	186.2	6.78	0
TYR	163.2	6.46	0
VAL	99.07	5.86	0

Supplementary Table 2. Amino acid parameters taken from Ref. [9].

Particle	Mass (g/mol)	Charge (e)
DNA ellipsoid	650	0
DNA phosphate	10^{-6}	-1

Supplementary Table 3. Chemically-specific model DNA parameters.

Interaction	σ (Å)	ϵ (kcal/mol)	λ
DNA ellipsoid–amino acid	8	0.01	1
DNA phosphate–amino acid	4	0.1	1

Supplementary Table 4. Chemically-specific model DNA–amino acid interaction parameters.

Interaction pair	ϵ (kcal/mol)	σ (Å)	r_c (Å)
Core–core	0.1	55	$2^{1/6}\sigma$
Core–DNA	$E(c)$	40	3σ
DNA–DNA	0.1	$S(c)$	$2^{1/6}\sigma$

Supplementary Table 5. Minimal model Lennard-Jones parameters. c is the salt concentration. $E(c)$ and $S(C)$ are linear interpolations of the data in Supplementary Table 6. Note that $r_c = 2^{1/6}\sigma$ implies that the potentials are repulsive only.

c (mol/L)	E(c) (kcal/mol)	S(c) (Å)
0.15	0.4	24
0.115	0.375	24.5
0.082	0.35	25
0.07	0.3	26
0.06	0.25	27
0.052	0.2	28
0.05	0.1	30
0.042	0.01	34

Supplementary Table 6. $E(c)$ and $S(c)$ in Supplementary Table 5 are found by linear interpolation of this data.

Parameter	Value
$\mathbf{S}_{\text{Core}}$	$\text{diag}(28, 28, 20)$
$\mathbf{S}_{\text{DNA}}$	$\text{diag}(12, 12, 12)$
ϵ	6 kcal/mol
r_c	5 Å
$\mathbf{E}_{\text{core}}$	$\text{diag}(1^{-1/\mu}, 1^{-1/\mu}, 0.0001^{-1/\mu})$
$\mathbf{E}_{\text{DNA}}$	$\text{diag}(1^{-1/\mu}, 1^{-1/\mu}, 1^{-1/\mu})$
μ	1
ν	1

Supplementary Table 7. Minimal model anisotropic potential parameters. diag(a,b,c) means diagonal 3x3 matrix with the elements a,b,c on the diagonal.

Replica	1	2	3	4	5	6	7	8	9	10	11	12	13	14	15	16
λ_D (Å)	8.00	8.34	8.70	9.07	9.46	9.86	10.29	10.73	11.19	11.67	12.16	12.69	13.23	13.79	14.38	15.00

Supplementary Table 8. HREMD Debye Length (λ_D) values.

Interaction	Parameter(s)	Value	Justification
Rigid Base-Pair (eq. 3)	$\mathbf{K}$	6x6 matrix for each of the 16 possible DNA base-pair steps	We use values from the Orozco group [6] which are available on the NAFlex web server [7]. These were computed from a large set of all-atom molecular dynamics simulations of DNA.
	$\boldsymbol{\phi}_0$	Different equilibrium values for each of the 16 possible DNA base-pair steps	
Protein bonds (eq. 2)	r_0	3.5 Å	Values from the Kim-Hummer protein model [9, 11] .
	k	$20\,\text{kcal/mol/Å}^2$	
Hydrophobic (eq. 5)	ϵ, σ, λ, r_c	Protein: Supplementary Table 2<br>DNA: Supplementary Table 4	Pure protein parameters taken from the Kim-Hummer model [9, 11].<br>DNA-Protein values are fitted to radial-distribution functions from our all-atom nucleosome simulations [1].
Electrostatic (eq. 4)	Charge	Protein: Supplementary Table 2<br>DNA: Supplementary Table 3	Charges are set to the intrinsic charge of the molecule, the Debye–Hückel approximation is reasonable in the monovalent salt ranges found in cells.
	κ	Salt-dependence via eq. 9	

Supplementary Table 9. Summary of chemically-specific model parameters.

Interaction	Parameter(s)	Value	Justification
Minimal RBP (eq. 12)	$\mathbf{K}_{\text{minimal}}$	6x6 diagonal matrix, see eq. 26	Fit to helical parameter distributions from chemically-specific coarse-grained simulations of 200 bp DNA strands.
	$\boldsymbol{\phi}_{\mathbf{0},\text{minimal}}$	(0,0,16.4Å,0,0,0)	
LJ (eq. 11)	σ, ϵ	Particle dependent, see Supplementary Table 5	Initial estimates obtained by comparison of internucleosome PMFs of the chemically-specific and minimal models. Salt-dependent values obtained by fitting the radius of gyration of 12-nucleosome chromatin arrays from the chemically-specific model to the minimal model.
	r_c	Particle dependent, see Supplementary Table 5	DNA-DNA and Core-Core interactions are repulsive only (same-sign charges), whilst DNA-Core interactions contain an attractive tail (opposite-sign charges).
Anisotropic (eq. 13)	$\mathbf{S}_{\text{Core}}$	$\text{diag}(28, 28, 20)$	Optimized to approximate the geometry and free energy profile of DNA–histone core binding, see Supplementary Figure 4c.
	$\mathbf{S}_{\text{DNA}}$	$\text{diag}(12, 12, 12)$	
	ϵ	6 kcal/mol	
	r_c	5 Å	
	$\mathbf{E}_{\text{core}}$	$\text{diag}(1^{-1/\mu}, 1^{-1/\mu}, 0.0001^{-1/\mu})$	
	$\mathbf{E}_{\text{DNA}}$	$\text{diag}(1^{-1/\mu}, 1^{-1/\mu}, 1^{-1/\mu})$	
	μ	1	
	ν	1	

Supplementary Table 10. Summary of minimal model parameters.

Supplementary Figures

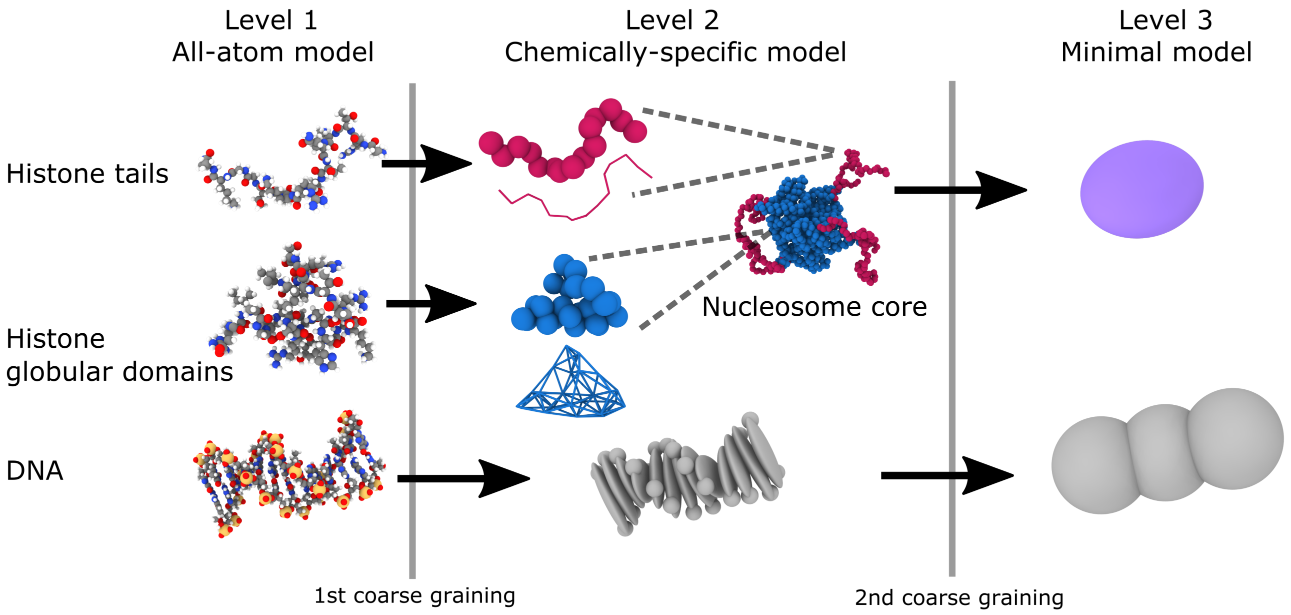

Supplementary Figure 1. Additional details on the multiscale coarse-grained mapping, complimentary to Figure 1 (main text). The three panels provide schematic representations of the different particles featured in our multiscale approach, which spans three levels of resolution. **(Level 1)** All-atom representations of histone tails, histone globular domains (GD), and DNA. **(Level 2)** Chemically-specific representations of histones, both tails and GD, with the difference in bond topology illustrated, and DNA. **(Level 3)** Minimal coarse-grained model showing the histone core and DNA mapping.

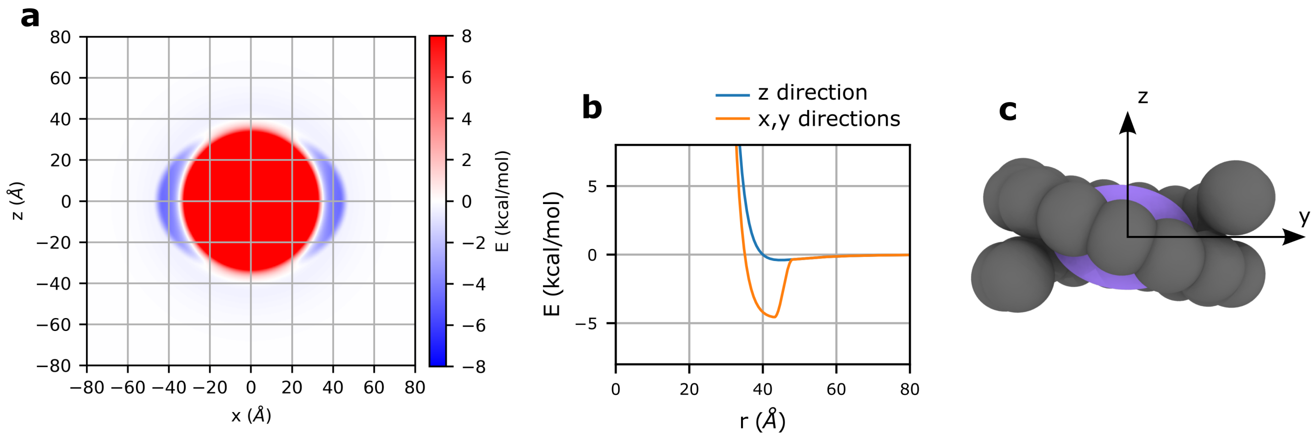

Supplementary Figure 2. Description of minimal model anisotropic potential. a,b Graphs of the potential acting on the DNA beads due to the core bead; this includes both the LJ and anisotropic potential. The potential has a strong attractive region around the nucleosome xy plane and weak attraction at the z poles. It is symmetric around the z axis. **c** Illustration of DNA binding to the attractive region of the anisotropic potential.

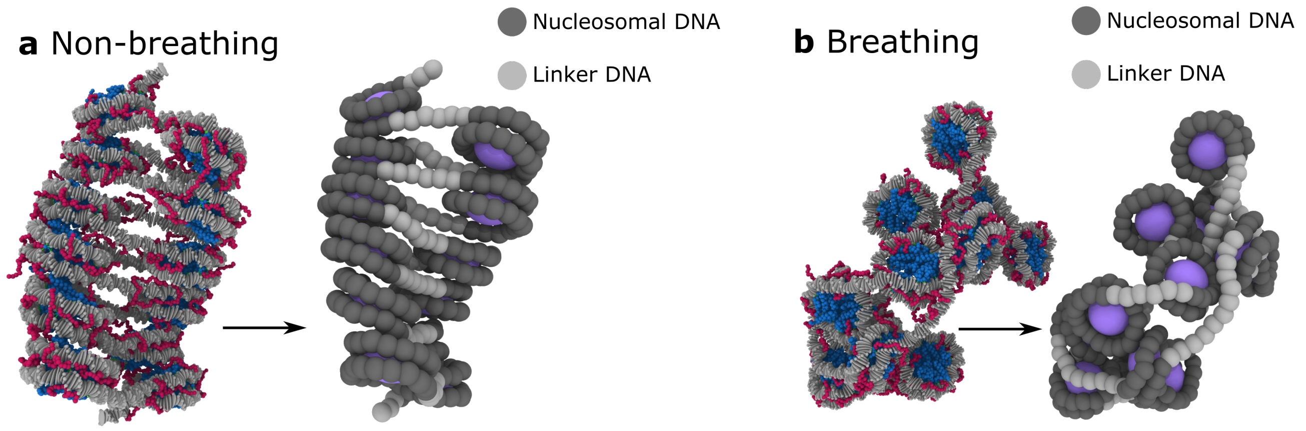

Supplementary Figure 3. Mapping of minimal model initial structures from chemically-specific model equilibrium structures. The important difference between the non-breathing (**a**) and the breathing (**b**) chromatin is the amount of linker DNA, at higher salt the non-breathing chromatin has significantly more.

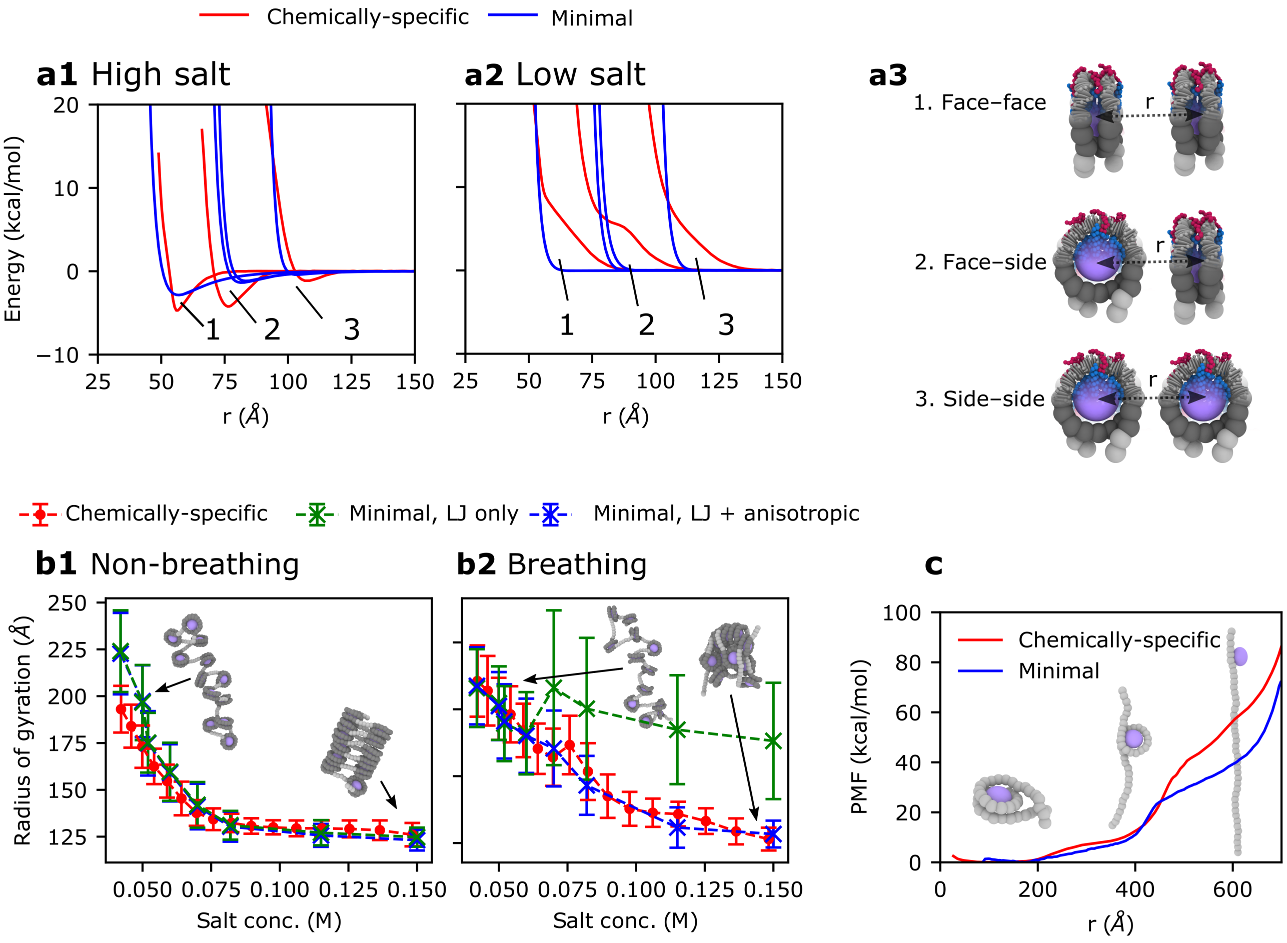

Supplementary Figure 4. Fitting of the minimal model potentials and parameters to the behavior of chromatin within the chemically-specific model. a Orientational-dependent interactions between two nucleosomes at high (0.15 M) and low (0.05 M) concentration of NaCl, **a1** and **a2** respectively, for the chemically specific (red) and minimal (blue) models. The chemically-specific model curves are PMFs computed using the center-to-center nucleosome distance among nucleosome pairs in the three different relative orientations described in **a3**. The minimal model curves represent the total pairwise energy as a function of center-to-center nucleosome distance in the three same orientations. **b** Salt-dependent radius of gyration of 12-nucleosome 165-bp chromatin for the chemically–specific model and the minimal model. The figure compares the values computed with and without the anisotropic potential for chromatin with non-breathing **b1** and breathing **b2** nucleosomes. This illustrates the need of the anisotropic potential to recover the behavior of chromatin with breathing nucleosomes at higher salt. The data points and corresponding error bars are the mean±s.d. obtained from n=500 independent configurations. **c** Nucleosome unwrapping PMFs for both models showing good agreement in the low extension regime.

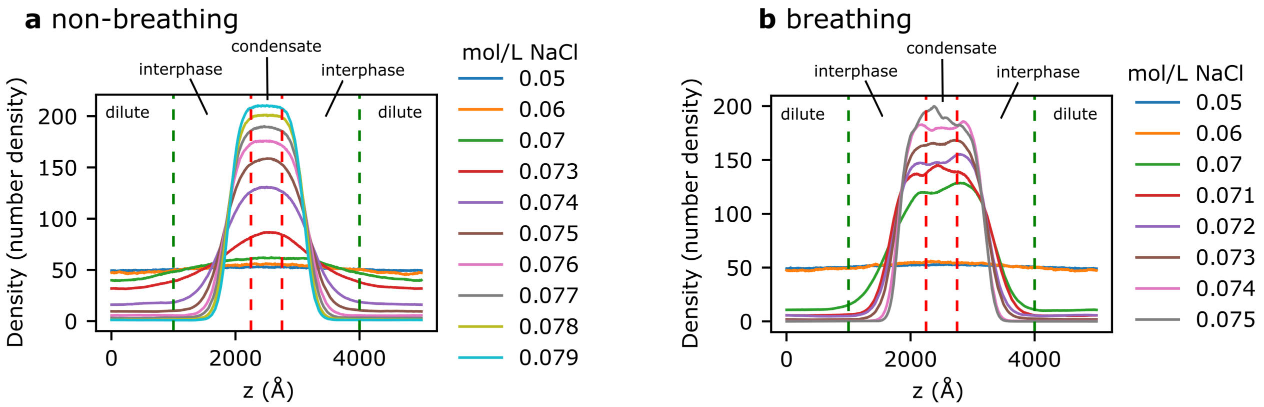

Supplementary Figure 5. Equilibration of simulations and determination of chromatin density. Density profiles along the box long axis z, of a phase-separated system obtained via direct coexistence simulations at varying salt concentrations for chromatin with (**a**) non-breathing and (**b**) breathing nucleosomes. The presence of sharp, non-fluctuating interfaces (the two regions between the green dashed lines and the red dashed lines) is indicative of phase separation. The density of the condensate (middle region enclosed by the two red dashed lines) and dilute phase (outer regions from the extremes of the box to the green dashed lines) are averaged to determine the respective chromatin densities and the uncertainty on the measured density are given by the standard deviation. The interface regions are excluded from these calculations.

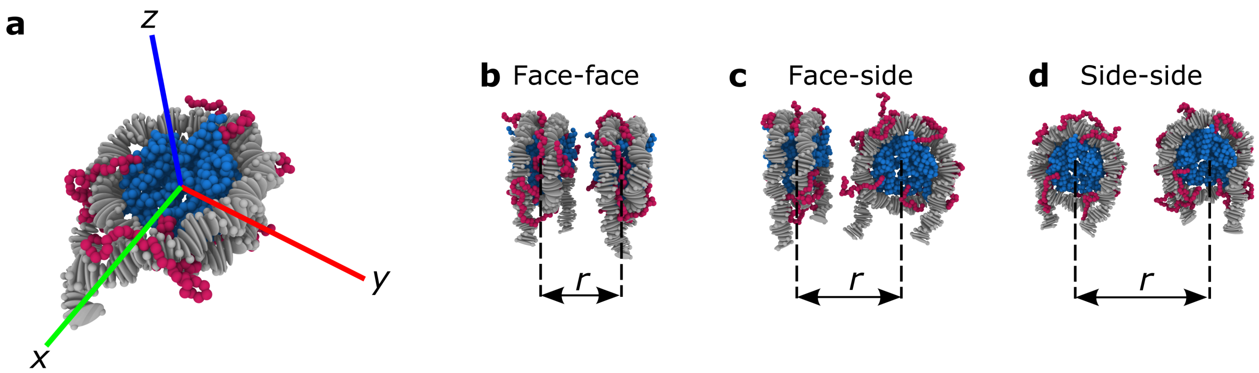

Supplementary Figure 6. Definition of nucleosome pair orientations. a Nucleosome orientation axis: x points from the center of the nucleosome to the dyad position, z points out of the top face, and $y = z \times x$. **b-d** Nucleosome–nucleosome interaction configurations, r is the center-to-center distance between two nucleosomes.

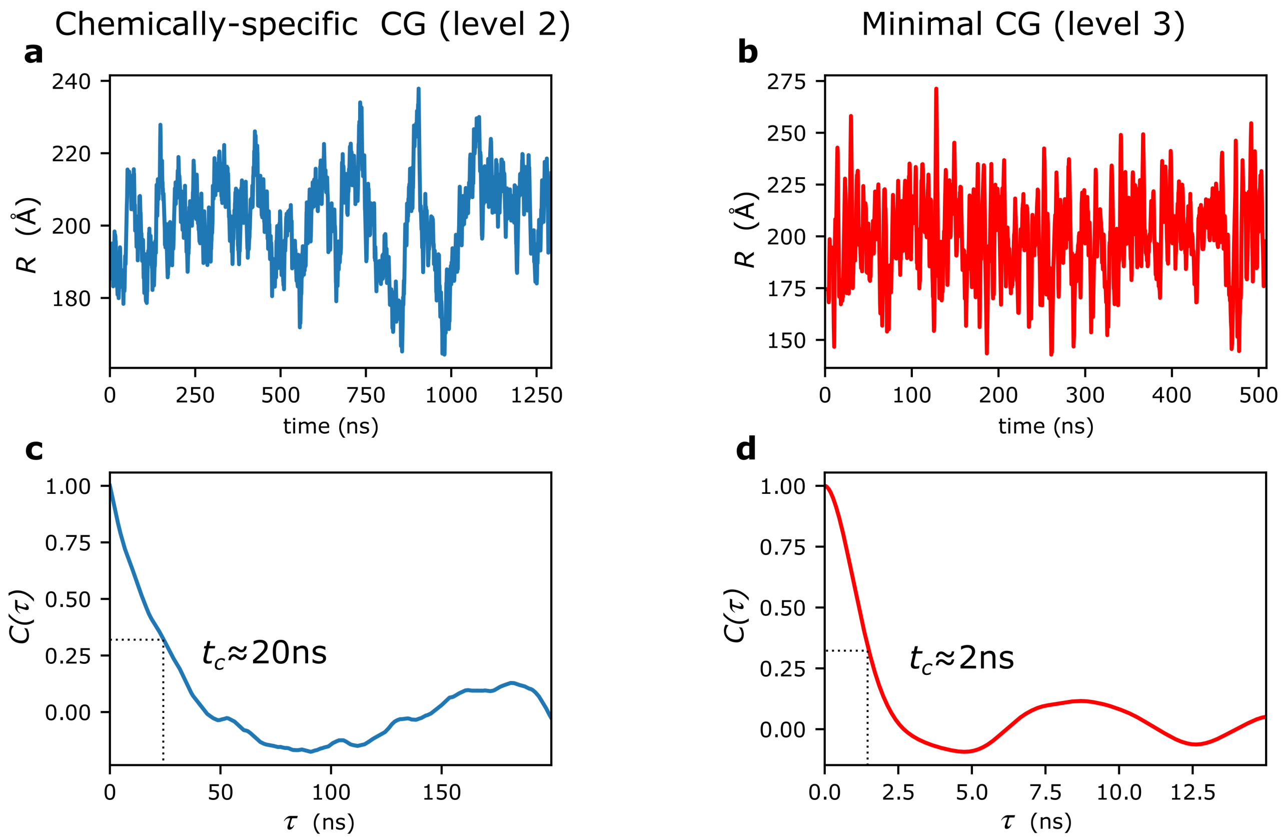

Supplementary Figure 7. Timescale comparison between chemically-specific and minimal models. a, b Time series of the radius of gyration R for 12-nucleosome chromatin using the chemically-specific model and the minimal model respectively. **c, d** Autocorrelation functions $C(\tau)$ of R for the chemically-specific model and minimal model respectively. The correlation time t_c is labeled; estimated from where the curves reach $1/e$ height.

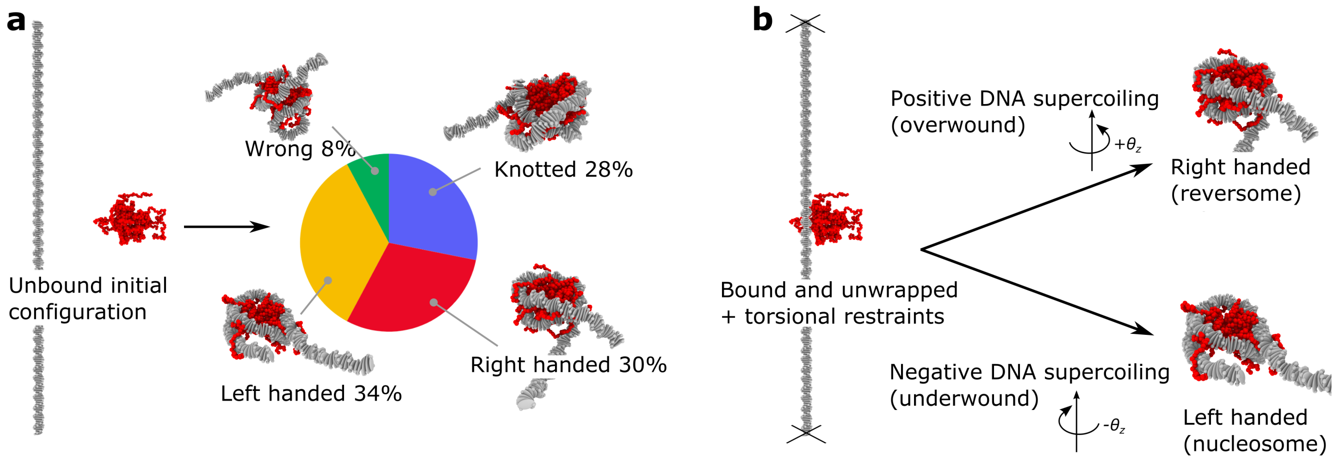

Supplementary Figure 8. Spontaneous formation of nucleosomes predicted by our chemically-specific coarse-grained model at 0.15 mol/L NaCl and 300 K. a Starting from an unbound DNA to histone core configuration, i.e., where the DNA and histone octamer are completely separated, as time progresses, the DNA wraps around the histone core in one of four possible ways: left-handed supercoiling (a canonical nucleosome) with a ∼34% probability, right-handed supercoiling (a reversesome) with a ∼30% probability, a knotted configuration which is neither a left-handed or right-handed supercoil but still resembles a nucleosome-like shape with a ∼28% probability, and other configurations which differ significantly from a nucleosome topology ("wrong") with a 8% probability. **b** Starting with a fully unwrapped nucleosome, but with the histone core bound to the DNA at a single point, we apply a weak super-coiling. In this case, as time progresses, the model always form nucleosomes when DNA is underwound, and reversomes when DNA is overwound.

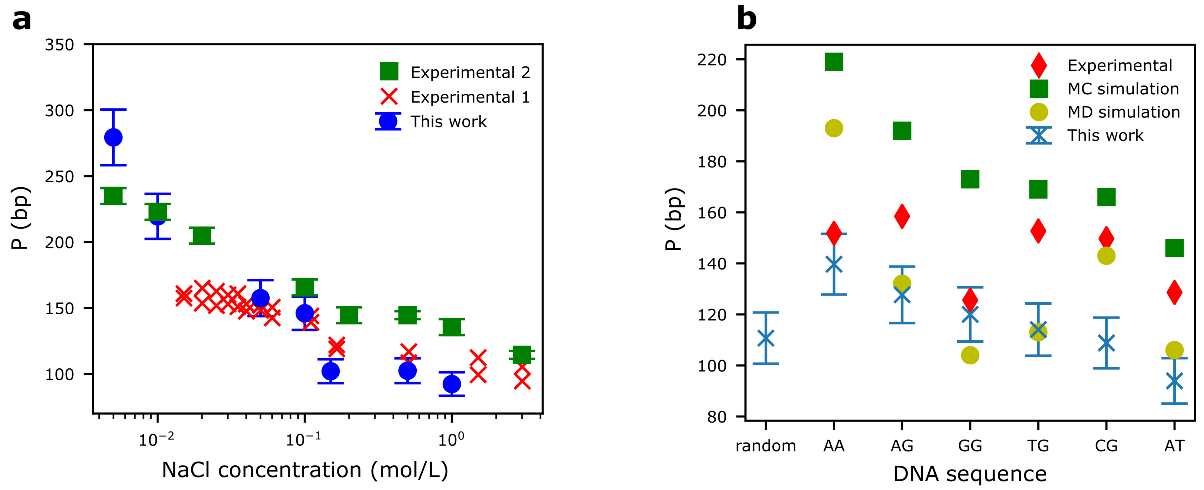

Supplementary Figure 9. Experimental validation of the modified DNA rigid base pair model with added phosphate charges. a Persistence length of DNA in units of base-pairs (bp) as a function of NaCl concentration in mol/L at 300K. Blue circles are the values obtained with our simulations on a set of 300 bp DNA strands with varying sequences. The data points are the value of P fitted by a non-linear least squares fit of equation 68, the error bars are the standard error in P reported by the least squares fitting method. 10 independent simulation trajectories were used. Red crosses (experimental 1) are values from single-molecule high-throughput tethered particle motion experiments on DNAs of 1201 and 2060 bp at room temperature [39]. Green squares (experimental 2) are values from Rayleigh light scattering experiments for a T7 bacteriophage DNA from [40]. **b** Persistence length of DNA as function of DNA sequence from our simulations (blue crosses) using a random sequence and six poly(XY) sequences for DNA of 300 bp in length. The data points are the value of P fitted by a non-linear least squares fit of equation 68, the error bars are the standard error in P reported by the least squares fitting method. 10 independent simulation trajectories were used. We compare to values from experimental cyclization assays [41], coarse-grain Monte Carlo simulations [42], and all-atom MD simulations [42].

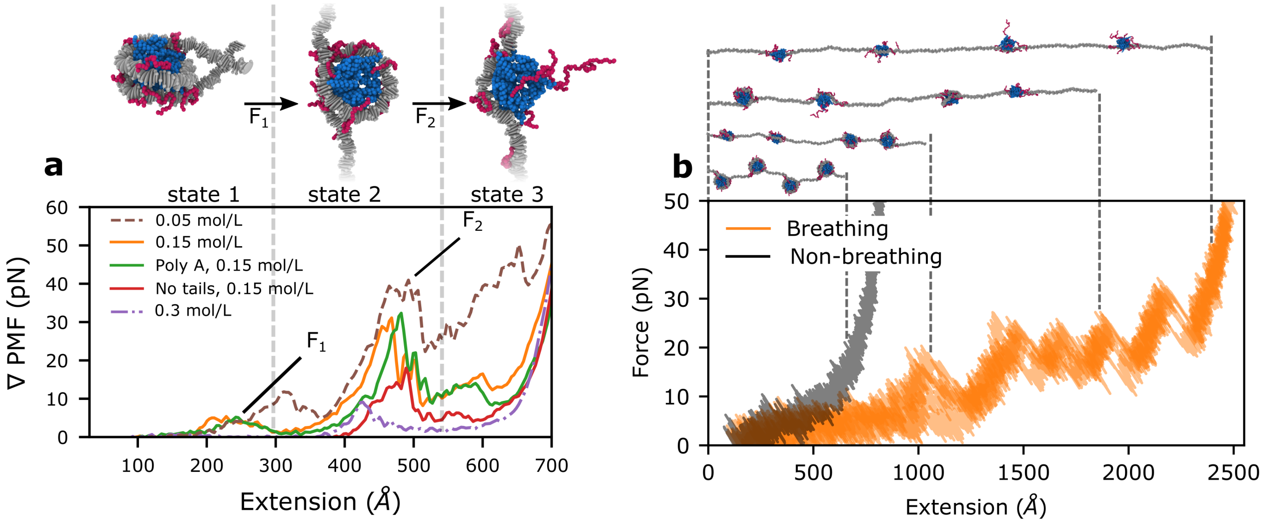

Supplementary Figure 10. Force-induced unwrapping behavior of mononucleosomes and chromatin under varying conditions with our chemically-specific coarse-grained model. **a** Model predictions for the force-induced unwrapping behavior of mononucleosomes under varying conditions. Top: Representative simulation snapshots of nucleosome configurations (color coded as in Figure 1 of the main–Level 2) at three different stages of the unwrapping process, showing a fully wrapped nucleosome (state 1) at low pulling forces ($\leq F_1$), a nucleosome with the first turn unwrapped (state 2) at intermediate forces (F_1–F_2), and a fully unwrapped nucleosome (state 3) at higher forces ($\geq F_2$). Bottom: Force (computed from the gradient of the PMF) in pN for nucleosome unwrapping as a function of the end-to-end DNA distance (or extension). The dashed brown, solid orange, and dashed purple curves correspond to 1KX5 DNA sequence [43] nucleosome simulations at 0.05 M, 0.15 M, and 0.3 M of NaCl, respectively. The green curve corresponds to simulations of a poly-A DNA sequence nucleosome at 0.15 M NaCl. The red curve was calculated for a nucleosome with all histone tails clipped at 0.15 M NaCl. **b** Force-extension for 4 nucleosome chromatin. There are 5 curves overlaid for chromatin with breathing nucleosomes (orange) and one curve using the fixed non-breathing version of the model for reference (black). Illustrated conformations are shown at the top with the extension indicated by the dashed lines.

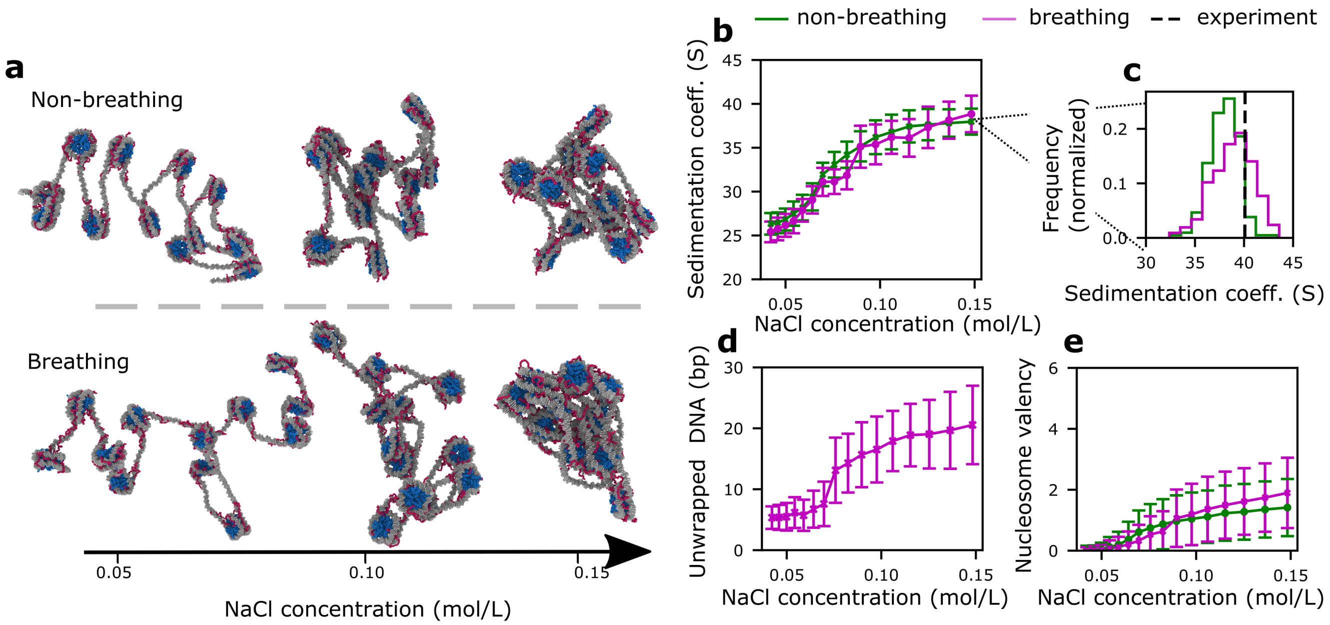

Supplementary Figure 11. 12-nucleosome 195 bp NRL chromatin simulations. **a** Representative simulation snapshots of 195-bp 12-nucleosome chromatin with non-breathing (top) versus breathing (bottom) nucleosomes at three different salt concentrations: 0.05 mol/L, 0.10 mol/L and 0.15 mol/L of NaCl. **b** Sedimentation coefficients versus NaCl concentration (right) for chromatin with non-breathing (green) versus breathing (magenta) nucleosomes. The data points and corresponding error bars are the mean±s.d. obtained from n=100 independent configurations. **c** Histograms comparing the distributions of sedimentation coefficient values for chromatin with non-breathing (green solid) and breathing (magenta solid) at 0.15 mol/L in our simulations with the experimental value from reference [44] (black dashed). **d** Number of average DNA bp that unwrap per nucleosome in our simulations at varying concentration of NaCl. The data points and corresponding error bars are the mean±s.d. obtained from n=100 independent configurations. **e** Average nucleosome valency versus concentration of NaCl. The data points and corresponding error bars are the mean±s.d. obtained from n=100 independent configurations.

Supplementary References

1. A. Sridhar, S. E. Farr, G. Portella, T. Schlick, M. Orozco, and R. Collepardo-Guevara, Proceedings of the National Academy of Sciences **117**, 7216 (2020).
2. S. Plimpton, Journal of Computational Physics **117**, 1 (1995).
3. W. K. Olson, A. A. Gorin, X. J. Lu, L. M. Hock, and V. B. Zhurkin, Proceedings of the National Academy of Sciences of the United States of America **95**, 11163 (1998).
4. F. Lankaš, J. Šponer, J. Langowski, and T. E. Cheatham, Biophysical Journal **85**, 2872 (2003).
5. A. Pérez, A. Noy, F. Lankas, F. J. Luque, and M. Orozco, Nucleic Acids Research **32**, 6144 (2004).
6. P. D. Dans, A. Pérez, I. Faustino, R. Lavery, and M. Orozco, Nucleic Acids Research **40**, 10668 (2012).
7. A. Hospital, I. Faustino, R. Collepardo-Guevara, C. González, J. L. Gelpí, and M. Orozco, Nucleic acids research **41**, W47 (2013).
8. X.-J. Lu, M. El Hassan, and C. Hunter, Journal of Molecular Biology **273**, 668 (1997).
9. G. L. Dignon, W. W. Zheng, Y. C. Kim, R. B. Best, and J. Mittal, Plos Computational Biology **14**, e1005941 (2018).
10. S. Miyazawa and R. L. Jernigan, Journal of Molecular Biology **256**, 623 (1996).
11. Y. C. Kim and G. Hummer, Journal of Molecular Biology **375**, 1416 (2008).
12. A. Fathizadeh, A. Berdy Besya, M. Reza Ejtehadi, and H. Schiessel, The European Physical Journal E **36**, 21 (2013).
13. X. Lu and W. K. Olson, Nucleic Acids Research **31**, 5108 (2003).
14. I. Bahar, A. R. Atilgan, and B. Erman, Folding and Design **2**, 173 (1997).
15. N. Grønbech-Jensen and O. Farago, Molecular Physics **111**, 983 (2013).
16. J. G. Gay and B. J. Berne, The Journal of Chemical Physics **74**, 3316 (1981).
17. I. R. Cooke, K. Kremer, and M. Deserno, Phys. Rev. E **72**, 011506 (2005).
18. W. M. Brown, M. K. Petersen, S. J. Plimpton, and G. S. Grest, The Journal of Chemical Physics **130**, 044901 (2009).
19. M. P. Allen and G. Germano, Molecular Physics **104**, 3225 (2006).
20. J. Lequieu, A. Córdoba, D. C. Schwartz, and J. J. De Pablo, ACS Central Science **2**, 660 (2016).
21. G. Fiorin, M. L. Klein, and J. Hénin, Molecular Physics **111**, 3345 (2013).
22. A. Grossfield, "Wham: the weighted histogram analysis method, version 2.0.9," .
23. J. R. Espinosa, E. Sanz, C. Valeriani, and C. Vega, Journal of Chemical Physics **139**, 144502 (2013).
24. R. García Fernández, J. L. F. Abascal, and C. Vega, Journal of Chemical Physics **124**, 144506 (2006).
25. A. Ladd and L. Woodcock, Chemical Physics Letters **51**, 155 (1977).
26. J. R. Espinosa, A. Garaizar, C. Vega, D. Frenkel, and R. Collepardo-Guevara, Journal of Chemical Physics **150**, 224510 (2019).
27. P. J. Fleming and K. G. Fleming, Biophysical Journal **114**, 856 (2018).
28. P. Pfaffle and V. Jackson, Journal of Biological Chemistry **265**, 16821 (1990).

29. A. Bancaud, G. Wagner, N. e Silva, C. Lavelle, H. Wong, J. Mozziconacci, M. Barbi, A. Sivolob, E. Le Cam, L. Mouawad, J. L. Viovy, J. M. Victor, A. Prunell, N. Conde e Silva, C. Lavelle, H. Wong, J. Mozziconacci, M. Barbi, A. Sivolob, E. Le Cam, L. Mouawad, J. L. Viovy, J. M. Victor, and A. Prunell, Molecular Cell **27**, 135 (2007).

30. C. Lavelle, P. Recouvreux, H. Wong, A. Bancaud, J. L. Viovy, A. Prunell, and J. M. Victor, Cell **139**, 1216 (2009).

31. T. Furuyama and S. Henikoff, Cell **138**, 104 (2009).

32. G. Lamour, J. B. Kirkegaard, H. Li, T. P. Knowles, and J. Gsponer, Source Code for Biology and Medicine **9**, 16 (2014).

33. P. Cifra, Polymer **45**, 5995 (2004).

34. H.-P. Hsu, W. Paul, and K. Binder, Macromolecules **43**, 3094 (2010).

35. S. Mihardja, A. J. Spakowitz, Y. Zhang, and C. Bustamante, Proceedings of the National Academy of Sciences of the United States of America **103**, 15871 (2006).

36. M. Kruithof and J. Van Noort, Biophysical Journal **96**, 3708 (2009).

37. H. Meng, K. Andresen, and J. Van Noort, Nucleic Acids Research **43**, 3578 (2015).

38. M. L. Bennink, S. H. Leuba, G. H. Leno, J. Zlatanova, B. G. De Grooth, and J. Greve, Nature Structural Biology **8**, 606 (2001).

39. A. Brunet, C. Tardin, L. Salomé, P. Rousseau, N. Destainville, and M. Manghi, Macromolecules **48**, 3641 (2015).

40. E. S. Sobel and J. A. Harpst, Biopolymers **31**, 1559 (1991).

41. S. Geggier and A. Vologodskii, Proceedings of the National Academy of Sciences **107**, 15421 (2010).

42. J. S. Mitchell, J. Glowacki, A. E. Grandchamp, R. S. Manning, and J. H. Maddocks, Journal of Chemical Theory and Computation **13**, 1539 (2017).

43. C. A. Davey, D. F. Sargent, K. Luger, A. W. Maeder, and T. J. Richmond, Journal of Molecular Biology **319**, 1097 (2002).

44. S. J. Correll, M. H. Schubert, and S. A. Grigoryev, EMBO Journal **31**, 2416 (2012).
