# Supplementary material for: Nucleosome plasticity is a critical element of chromatin liquid–liquid phase separation and multivalent nucleosome interactions
Source: Nat Commun. 2021 May 17;12:2883. doi: 10.1038/s41467-021-23090-3 (PMC8129070; doi:10.1038/s41467-021-23090-3)
Supplement: Supplementary file 3 — Reporting Summary [file 41467_2021_23090_MOESM3_ESM.pdf]

nature research

Corresponding author(s): Rosana Collepardo-Guevara

Last updated by author(s): 22/02/2021

Reporting Summary

Nature Research wishes to improve the reproducibility of the work that we publish. This form provides structure for consistency and transparency in reporting. For further information on Nature Research policies, see our Editorial Policies and the Editorial Policy Checklist.

Statistics

For all statistical analyses, confirm that the following items are present in the figure legend, table legend, main text, or Methods section.

n/a	Confirmed	
☐	☒	The exact sample size (n) for each experimental group/condition, given as a discrete number and unit of measurement
☒	☐	A statement on whether measurements were taken from distinct samples or whether the same sample was measured repeatedly
☒	☐	The statistical test(s) used AND whether they are one- or two-sided<br>*Only common tests should be described solely by name; describe more complex techniques in the Methods section.*
☒	☐	A description of all covariates tested
☒	☐	A description of any assumptions or corrections, such as tests of normality and adjustment for multiple comparisons
☐	☒	A full description of the statistical parameters including central tendency (e.g. means) or other basic estimates (e.g. regression coefficient) AND variation (e.g. standard deviation) or associated estimates of uncertainty (e.g. confidence intervals)
☒	☐	For null hypothesis testing, the test statistic (e.g. F, t, r) with confidence intervals, effect sizes, degrees of freedom and P value noted<br>*Give P values as exact values whenever suitable.*
☒	☐	For Bayesian analysis, information on the choice of priors and Markov chain Monte Carlo settings
☒	☐	For hierarchical and complex designs, identification of the appropriate level for tests and full reporting of outcomes
☒	☐	Estimates of effect sizes (e.g. Cohen's d, Pearson's r), indicating how they were calculated

Our web collection on statistics for biologists contains articles on many of the points above.

Software and code

Policy information about availability of computer code

Data collection	All our simulations were performed in LAMMPS (version 3rd March 2020), which is a free and open-source software, distributed under the terms of the GNU General Public License. Our costume code has been deposited here: https://doi.org/10.6084/m9.figshare.13663685.v1
Data analysis	Analyses were performed with LAMMPS (version 3rd March 2020), 3DNA (version 2.3), python (version 3.8.5) with NumPy (version 1.19.2) and SciPy (version 1.5.2) and the WHAM program (version 2.0.9). For visualization we use Ovito (version 3.0.0). All this software is free and open-source.

For manuscripts utilizing custom algorithms or software that are central to the research but not yet described in published literature, software must be made available to editors and reviewers. We strongly encourage code deposition in a community repository (e.g. GitHub). See the Nature Research guidelines for submitting code & software for further information.

Data

Policy information about availability of data

All manuscripts must include a data availability statement. This statement should provide the following information, where applicable:

- Accession codes, unique identifiers, or web links for publicly available datasets
- A list of figures that have associated raw data
- A description of any restrictions on data availability

All data needed to reproduce our work is available in the manuscript, supporting information, and in https://doi.org/10.6084/m9.figshare.13663859.v1

Field-specific reporting

Please select the one below that is the best fit for your research. If you are not sure, read the appropriate sections before making your selection.

- [x] Life sciences
- [ ] Behavioural & social sciences
- [ ] Ecological, evolutionary & environmental sciences

For a reference copy of the document with all sections, see nature.com/documents/nr-reporting-summary-flat.pdf

Life sciences study design

All studies must disclose on these points even when the disclosure is negative.

Sample size	Our analyzes use a sample size of n=500 independent configurations output from our simulations after convergence.
Data exclusions	No data was excluded
Replication	Our simulations use an advanced enhanced sampling protocol with 16 Debye-length replicas per simulation.
Randomization	Data randomization was not required as we compare with a control of chromatin with non-breathing nucleosomes.
Blinding	Data blinding was not required as we compare with a control of chromatin with non-breathing nucleosomes.

Reporting for specific materials, systems and methods

We require information from authors about some types of materials, experimental systems and methods used in many studies. Here, indicate whether each material, system or method listed is relevant to your study. If you are not sure if a list item applies to your research, read the appropriate section before selecting a response.

Materials & experimental systems

n/a	Involved in the study	
[x]	[ ]	Antibodies
[x]	[ ]	Eukaryotic cell lines
[x]	[ ]	Palaeontology and archaeology
[x]	[ ]	Animals and other organisms
[x]	[ ]	Human research participants
[x]	[ ]	Clinical data
[x]	[ ]	Dual use research of concern

Methods

n/a	Involved in the study	
[x]	[ ]	ChIP-seq
[x]	[ ]	Flow cytometry
[x]	[ ]	MRI-based neuroimaging
